# Rapid, reference-free identification of bacterial pathogen transmission using optimized split k-mer analysis

**DOI:** 10.1099/mgen.0.001347

**Published:** 2025-03-06

**Authors:** Christopher H. Connor, Charlie K. Higgs, Kristy Horan, Jason C. Kwong, M. Lindsay Grayson, Benjamin P. Howden, Torsten Seemann, Claire L. Gorrie, Norelle L. Sherry

**Affiliations:** 1 Department of Microbiology & Immunology at the Peter Doherty Institute for Infection & Immunity, University of Melbourne, Melbourne, Victoria, Australia; 2 Microbiological Diagnostic Unit (MDU) Public Health Laboratory, Department of Microbiology & Immunology at the Peter Doherty Institute for Infection & Immunity, University of Melbourne, Melbourne, Victoria, Australia; 3 Department of Infectious Diseases & Immunology, Austin Health, Heidelberg, Victoria, Australia; 4 Centre for Pathogen Genomics, University of Melbourne, Melbourne, Victoria, Australia

**Keywords:** bacterial genomics, infection prevention and control, *k*-mer-based methodology, pathogen transmission

## Abstract

Infections caused by multidrug-resistant organisms (MDROs) are difficult to treat and often life threatening and place a burden on the healthcare system. Minimizing the transmission of MDROs in hospitals is a global priority with genomics proving to be a powerful tool for identifying the transmission of MDROs. To optimize the utility of genomics for prospective infection control surveillance, results must be available in real time, reproducible and simple to communicate to clinicians. Traditional reference-based approaches suffer from several limitations for prospective genomic surveillance. Whilst reference-free or pairwise genome comparisons avoid some of these limitations, they can be computationally intensive and time consuming. Split *k*-mer analysis (SKA) offers a viable alternative facilitating rapid reference-free pairwise comparisons of genomic data, but the optimum SKA parameters for the detection of transmission have not been determined. Additionally, the accuracy of SKA-based inferences has not been measured, nor whether modified quality control parameters are required. Here, we explore the performance of 60 SKA parameter combinations across 50 simulations to quantify the false negative and positive SNP proportions for *Escherichia coli*, *Enterococcus faecium*, *Klebsiella pneumoniae* and *Staphylococcus aureus*. Using the optimum parameter combination, we explore concordance between SKA, multilocus sequence typing (MLST), core genome MLST (cgMLST) and Snippy in a real-world dataset. Lastly, we investigate whether simulated plasmid gain or loss could impact SNP detection with SKA. This work identifies that the use of SKA with sequencing reads, a *k*-mer length of 19 and a minor allele frequency filter of 0.01 is optimal for MDRO transmission detection. Whilst SNP detection with SKA (when used with sequencing reads) undercalls SNPs compared to Snippy, it is significantly faster, especially with larger datasets. SKA has excellent concordance with MLST and cgMLST and is not impacted by simulated plasmid movement. We propose that the use of SKA for the detection of bacterial pathogen transmission is superior to traditional methodologies, capable of providing results in a much shorter timeframe.

**Impact Statement**

Transmission of bacterial pathogens in hospitals is of great concern for patient welfare. Genomics can provide us with greater power to identify potential outbreaks of bacterial pathogens. To be effective, hospitals require genomic analysis to be rapid and consistent; traditional methodologies are not capable of meeting these requirements. Split *k*-mer analysis (SKA) is emerging as a viable alternative, capable of rapidly providing consistent results. However, the accuracy and best data practices for SKA analysis have not been established. Here, we explore an extensive set of parameter combinations to determine the optimum conditions for transmission detection with SKA. We provide accurate quantification of the error rate for SKA, as well as its concordance with other methods. We propose that SKA aligns well with traditional typing and clustering methods, and whilst the results of SKA do not precisely match pairwise read-mapping methods, SKA’s speed is superior. The ability to rapidly produce consistent results that align with traditional typing methods makes SKA suitable for outbreak surveillance of bacterial pathogens. The data we present here provide the foundation for best data practices for the implementation of SKA.

## Data Summary

This study used a simulated dataset that has been made available at https://doi.org/10.26188/c.7418404. This study also makes use of publicly available data from the BioProject: PRJNA565795. The authors confirm that all supporting data, code and protocols have been provided within the article or through supplementary data files.

## Introduction

Multidrug-resistant organisms (MDROs) pose a serious threat to public health. The World Health Organization has identified several pathogens that pose particular concern including *Escherichia coli*, *Enterococcus faecium*, *Klebsiella pneumoniae* and *Staphylococcus aureus* [1,2]. These organisms present a serious burden for hospitals and are associated with increased patient morbidity and mortality [3,4]. Identifying the transmission of these pathogens in clinical environments is a priority for tackling their spread and improving patient outcomes [5]. Whole-genome sequencing has greatly improved our ability to identify outbreaks (including in healthcare settings) and, to some degree, has revolutionized pathogen surveillance [6]. However, the optimal analysis method for identifying transmission by routine genomic surveillance is currently unclear.

**Fig. 1.**
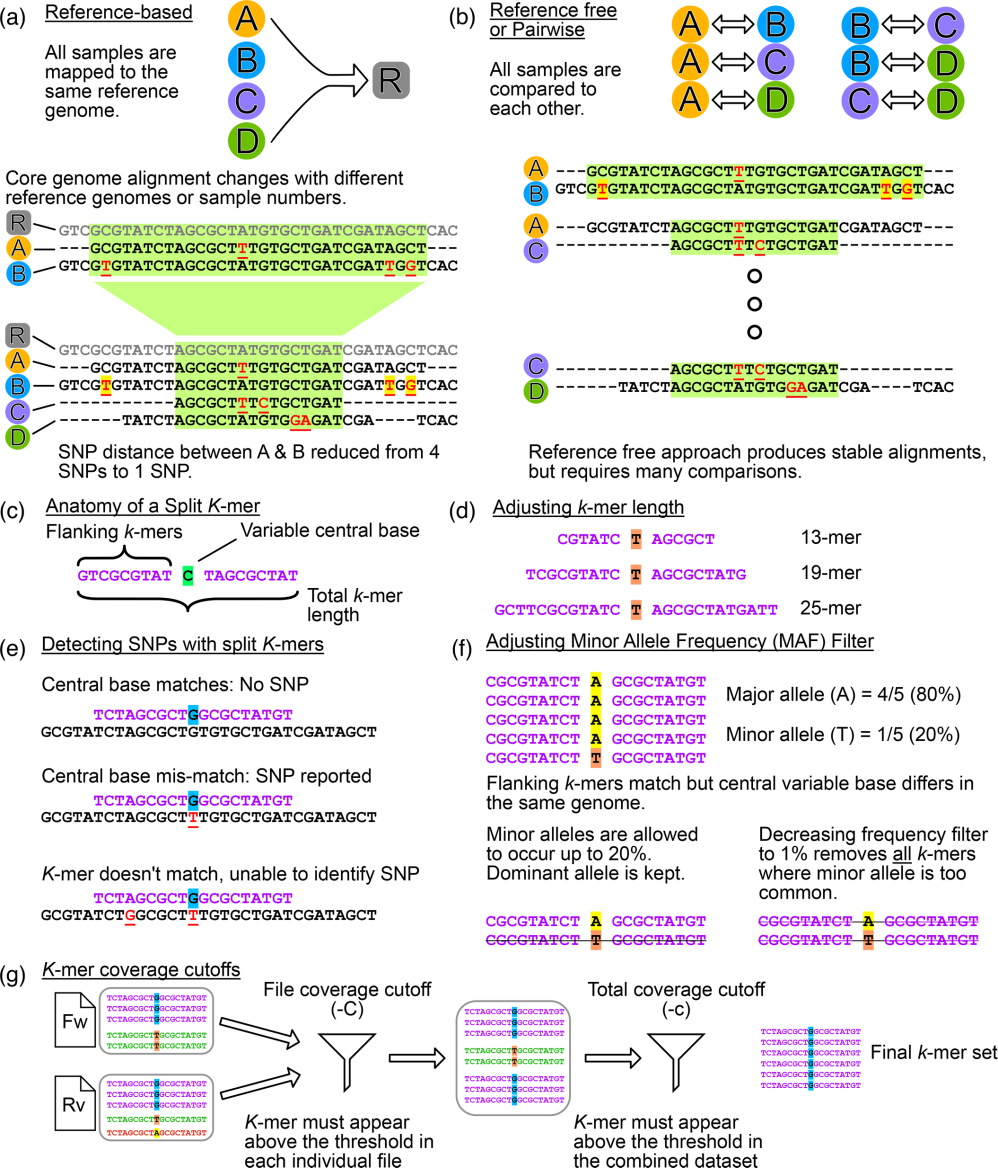
Diagrammatic explanation of differences between reference-based and reference-free genome comparison methods and specifics of split *k*-mer analysis. (a) Diagram of reference-based approach. Bacterial samples (circles labelled A through D) are mapped to a common reference genome. The resulting core genome alignment (highlighted in green) is used to calculate the SNP distance between samples. The inclusion of additional samples causes the core genome to shrink due to increased population diversity. This changes the SNP distance between isolates A and B as three SNPs are no longer part of the core genome (highlighted in yellow). (b) Diagram of reference-free/pairwise comparison methods which produce stable genome comparisons. Each bacterial sample is compared to every other sample. Comparisons can be performed with traditional read-mapping software or *k*-mer-based approaches. The number of comparisons that must be performed increases quadratically with sample size. (c) Anatomy of the split *k*-mer approach used by SKA. Two *k*-mers flank a central base that is allowed to vary. (d) Adjusting the *k*-mer length increases or decreases the size of the flanking *k*-mers. (e) The central base of the split *k*-mers is used to detect SNPs. *K*-mers must match exactly for SNP detection, preventing detection of SNPs less than the *k*-mer length apart. (f) The MAF filter parameter controls how SKA handles allelic variation in the sample. Sites with identical flanking *k*-mers where the central base is inconsistent are filtered to keep the dominant allele. A filter of 0.2 (default) allows up to 20% of the *k*-mers to contain the minor allele, and only the dominant allele is kept. If the filter is decreased, or the minor allele occurs at a higher frequency, then all *k*-mers are discarded. (g) *K*-mer coverage cutoffs in SKA remove *k*-mers that occur below the specified threshold in each individual input file (-C parameter, default 2) and below the specified threshold for the combined dataset (-c parameter, default 4).

Effective genomic surveillance must be rapid, accurate and consistent. In the healthcare setting, identification of pathogen transmission events must occur within a timeframe that allows for infection prevention control (IPC) measures to be implemented, minimizing further transmission and infections. Transmission events must be identified accurately to avoid costly and unnecessary IPC interventions, and they must also be consistent, allowing for clear outbreak identification and communication with clinicians. There are several methodologies for identifying bacterial transmission events; one of the most common is short-read mapping to a reference genome to identify SNPs [5]. This technique produces a core genome alignment (DNA that is common to all isolates), thereby excluding any variation in the accessory genome. SNP distances can then be calculated from the core genome alignment; a distance threshold then identifies pairs of isolates that are closely related and represent potential transmission events. This approach has several limitations that have been extensively characterized [7]. Firstly, the chosen reference genome greatly affects the results, with a more distantly related reference genome underestimating SNP distances; therefore, some *a priori* grouping of samples is necessary [such as by multilocus sequence typing (MLST)] with each group then analysed discretely with its own reference genome. Secondly, results are not stable over time; as new samples are added to the analysis, the resulting SNP distances can change – due to changing core genome alignments – creating problems with consistent outbreak definitions (Fig. 1a). Lastly, both the pre-grouping of samples and reference genome selection are not easily automated, leading to an ever-increasing manual workload, especially in the context of continual genomic surveillance.

These limitations can be eliminated by implementing a reference-free approach (Fig. 1b). Instead of mapping to a single shared reference genome, every isolate is compared directly to every other isolate in a pairwise fashion, resulting in a direct SNP distance measurement. This eliminates the concept of a shared core genome as each pairwise comparison will consider all common sequences between the two isolates, including the accessory genome such as plasmids. Traditional read-mapping pipelines such as Snippy (https://github.com/tseemann/snippy) can be used to perform the comparisons. This approach eliminates the need to pre-group the isolates, does not require a closely related reference genome and produces stable SNP distances [7]. However, as the number of samples grows, the number of pairwise comparisons to be performed grows quadratically. Using a read-mapping pipeline, such as Snippy, can lead to significant increases in analysis time. Faster analysis pipelines such as *k*-mer-based tools offer a viable alternative.

*K*-mer-based methodologies, such as split *k*-mer analysis (SKA) [8], have been explored as alternatives to read mapping for performing direct genome comparisons [9,10]. *K*-mer-based tools convert sequence data (either sequencing reads or genome assemblies) into short DNA sequences of length *k* (called *k*-mers), which are stored in a machine-readable format. This permits these tools to perform genome comparisons much faster and use a smaller amount of computing resources compared to read mapping. SKA differs from several other *k*-mer tools in that it uses split *k*-mers, where two *k*-mers of equal length flank a single base that is allowed to vary (Fig. 1c). This allows SKA to identify SNPs between two samples (Fig. 1e); SKA can calculate this SNP distance without constructing a genome alignment (alignment free), further facilitating rapid comparisons. The speed with which SKA can calculate SNPs makes it an attractive methodology for performing reference-free pairwise comparisons.

SKA can process sequencing reads (in fastq format) or genome assemblies (in fasta format), and both modes have adjustable analysis parameters. Two key parameters are *k*-mer length (Fig. 1d) and the minor allele frequency (MAF) filter (Fig. 1f), both of which affect SKA’s ability to detect SNPs. It is currently unclear which input type and combination of parameters produce the optimum results for detecting the transmission of bacterial pathogens, nor is it known how SKA performs at varying sequencing depths, and if additional data quality control steps are needed. Here, we aim to identify the optimum parameters for using SKA to detect the transmission of *E. coli*, *E. faecium*, *K. pneumoniae* and *S. aureus* (including input types, parameter combinations and sequencing depth), to allow confident use of SKA for genomic surveillance of pathogen transmission.

## Methods

### Synthetic dataset analysis with SKA

**Fig. 2.**
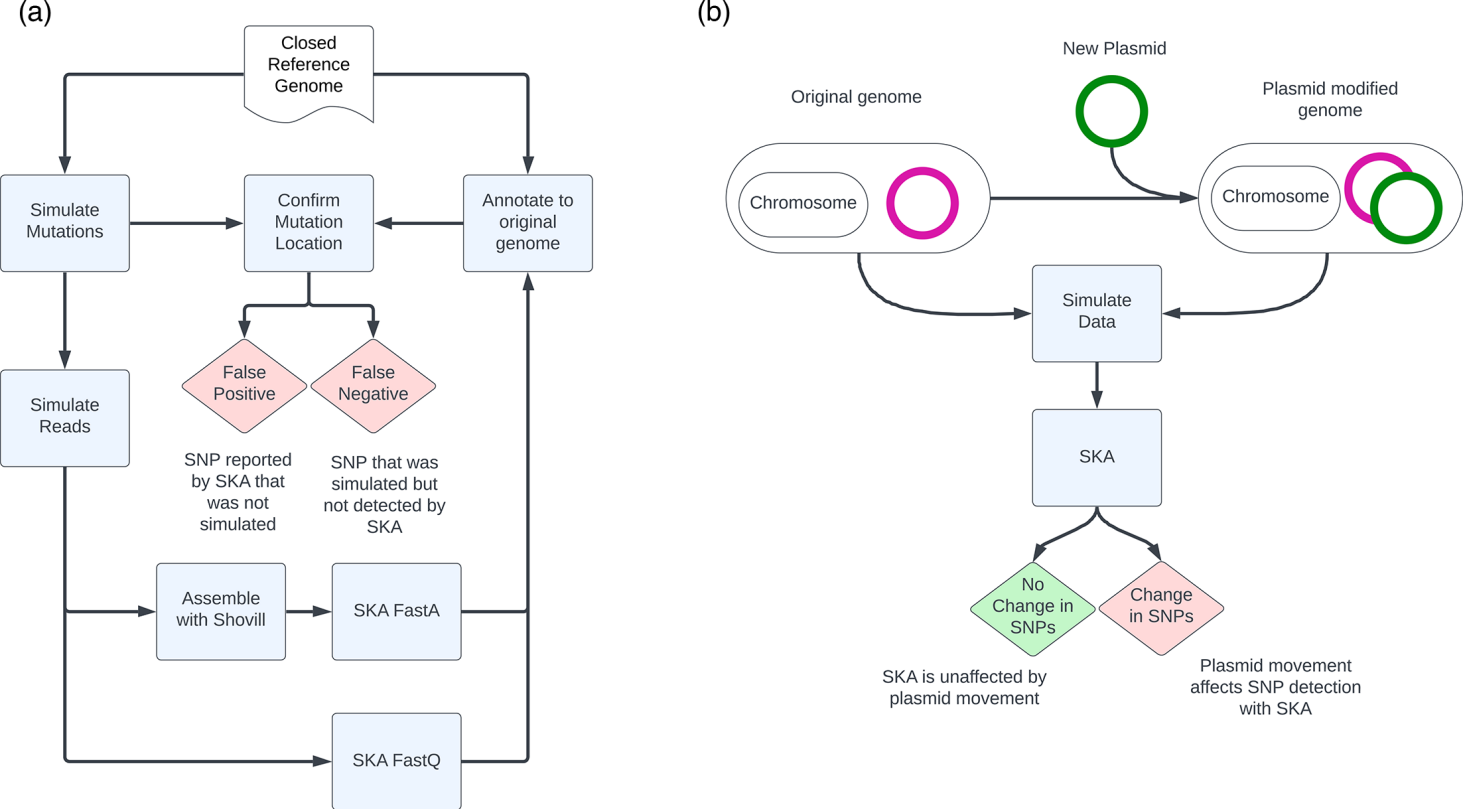
Methodology schematics. Diagram outlining methodological workflow for ascertaining the false negative and false positive percentages of SKA (**a**). Diagram outlining the methodological workflow for determining if plasmid gain/loss would affect SKA SNP detection (**b**).

To determine the optimum parameters for SKA, we generated a synthetic dataset of common MDROs with known (simulated) mutations (Tables 1 and S1, available in the online Supplementary Material and https://doi.org/10.26188/c.7418404). We downloaded closed reference genome assemblies for *E. coli* (GCA_000005845.2_ASM584v2), *E. faecium* (GCA_009734005.2_ASM973400v2), *K. pneumoniae* (GCA_000240185.2_ASM24018v2) and *S. aureus* (GCA_000013425.1_ASM1342v1) from the NCBI. Mutations were simulated in these reference genomes using Mutation-Simulator.py v2.0.3 [11]. SNPs were simulated at rates of 0.1, 1, 10 and 100 SNPs per kb. In addition, one simulation introduced SNPs at a rate of 0.1 SNP per kb alongside insertions, deletions, inversions, duplications and translocations all at a frequency of 0.1 per kb and all with a maximum length of 100 bases. Short-read data were simulated from each of the original reference genomes and their five mutated counterparts with ART Illumina v2.5.8 [12], using internal error profiles. Varying average read depth was simulated from 20- to 200-fold depth in increments of 20. A set of error-free reads was also simulated at 200-fold depth. The resulting reads were all trimmed with fastp v0.23.2 using the parameters cut_front, 3; sliding window size, 4; quality, 20; and minimum length, 36 [13]. Genome assemblies were made with Shovill v1.1.0 (https://github.com/tseemann/shovill) with a minimum contig length of 200 and default parameters. SKA was performed using SKA v1.0 [8]. The SKA fastq and fasta modules were used with total *k*-mer lengths of 13, 19, 25, 31 (default), 37 and 43, inclusive of the variable central base. These lengths correspond to command line parameters of 6, 9, 12, 15 (default), 18 and 21. The SKA fastq module was also tested with an MAF filter of 0.01, 0.1 and 0.2 at each *k*-mer length, as well as *k*-mer coverage cutoffs (file coverage and total coverage) or 1 and 2, 2 and 4 and 4 and 8. *K*-mers were mapped back to the reference genome with SKA annotate outputting only variant sites. Variant Call Format (VCF) files produced by SKA annotate and Mutation-Simulator.py were compared using pybedtools [14,15]. SNPs were classified based on Table 2. An overview of this methodology is provided in Fig. 2(a). We focussed on the percentage of SNPs that were misclassified (false positive or negative) by SKA. Typically, error rates would factor in the number of true negatives, which would dwarf the number of misclassifications. However, for the purposes of identifying highly similar genomes (transmission events), a small change in SNP identification – either through failure to detect or erroneous reporting – could dramatically impact an outbreak investigation.

**Table 1.** Reference genomes used for the creation of the simulated dataset, alongside parameters for mutation, sequencing coverage and SKA.

Genome	Accession	Size (Mbp)	GC%	SNP rate	Sequencing coverage	SKA with reads			SKA with assemblies
						*K*-mer	MAF filter	Coverage cutoff	*K*-mer
*E. coli*	GCF_000005845.2_ASM584v2	4.6	51	0.1, 1, 10, 100,0.1 with complex	20, 40, 60, 80, 100, 120,140, 160, 180, 200	13, 19, 25,31, 37, 43	0.01, 0.1, 0.2	1 and 2,2 and 4,4 and 8	13, 19, 25,31, 37, 43
*E. faecium*	GCF_009734005.2_ASM973400v2	2.9	38						
*K. pneumoniae*	GCF_000240185.2_ASM24018v2	5.7	57						
*S. aureus*	GCF_000013425.1_ASM1342v1	2.8	33						
**Totals**	**4**			**5**	**10**	**6**	**3**	**3**	**6**

**Table 2.** Classification of SNP error types

Type of error	Simulated mutation (Mutation-Simulator VCF)	Detected mutation (SKA VCF)	Percentage calculation
False positive	Missing	Present	$\frac{Number\;of\;SNPs\;unique\;to\;SKA}{Total\;number\;of\;SNPs\;identified\;by\;SKA}\times 100$
False negative	Present	Missing	$\frac{Number\;of\;SNPs\;unique\;to\;Mutation-Simulator}{Total\;number\;of\;Simulated\;SNPs}\times 100$
True positive	Present	Present	
True negative	Absent	Absent	

### Validation with a real-world dataset: comparison to MLST methods

To validate our observations made from our synthetic dataset, we used a real-world dataset collected as part of the ‘Controlling Superbugs project’, a prospective genomic surveillance study of four MDRO pathogens from hospitals in Victoria, Australia (PRJNA565795 [16]). We assessed how SNP distances were calculated with SKA compared with other genomic typing methodologies such as MLST and core genome MLST (cgMLST). All data were processed with fastp and Shovill using the same parameters as above. Sequence types were assigned using mlst v2.23.0 (https://github.com/tseemann/mlst) [17]. cgMLST allele calls were performed using chewBBACA v3.2.0 [18] with the schemes from cgmlst.org: *K. pneumoniae sensu lato* cgMLST v1.0 [19,20], *E. faecium* cgMLST v1.1 [21], *S. aureus* cgMLST v1.3 [22] and *E. coli* cgMLST v1.0 [20,23, 24]. Pairwise allelic differences were calculated using cgmlst-dists (https://github.com/tseemann/cgmlst-dists), and isolates were clustered using a single-linkage clustering algorithm with a 25 allelic difference threshold in python. Trimmed fastq files were analysed with SKA with a *k*-mer length of 19 and an MAF filter of 0.01, whilst a *k*-mer length of 37 was used for genome assemblies.

### Validation with a real-world dataset: comparison to pairwise Snippy

SNP distances between bacterial genomes are frequently calculated using Snippy. To validate our observations from the simulated dataset, we compared the SNP distances measured by SKA to Snippy. From the real-world dataset, we randomly selected 100 isolates per species that had an SKA distance of less than 100 SNPs. Pairwise Snippy comparisons were performed using Snippy v4.6.0 (https://github.com/tseemann/snippy). Within each isolate pair, *de novo* assemblies were used as reference genomes, and reads were mapped with a minimum fraction (minfrac) of 0.9 and a minimum coverage (mincov) of 10. Identified mutations were filtered to SNPs only.

### Comparison of computation time

To compare the analysis time required for Snippy and SKA to perform reference-free pairwise comparisons, we randomly selected isolates from our real-world dataset. Sample sizes used were 2, 4, 6, 8, 10, 15, 20, 25 and 50. For each sample size, pairwise comparisons were performed between all isolates. For analysis with Snippy, *de novo* genome assemblies were created with Shovill, with a minimum contig length of 200, 8 threads and default parameters. Isolates were randomly assigned as reference and query samples for Snippy mapping with a minfrac of 0.9, mincov of 10 and 8 threads. All isolates were processed with SKA-fastq module with a *k*-mer length of 19 and MAF filter of 0.01 before pairwise comparisons were performed with the SKA distance module. Analysis time was recorded with python. All analyses were performed on a 72 CPU (all Intel Xeon E5-2699 v3 running at 2.30 GHz), 377 GB RAM computer running Red Hat Enterprise Linux 8.9.

### Simulated plasmid movement

Pairwise comparisons with SKA include the accessory genome, such as plasmids. We investigated whether plasmid gain or loss could impact SNP detection with SKA. To test this, reference genome assemblies in Table 3 were downloaded from the NCBI. Plasmid and chromosome contigs were manually moved between assembly files as described in Table 3. The plasmid altered and original genome assemblies were used to simulate short-read data as described above (Table S2 and https://doi.org/10.26188/c.7418404). The resulting reads were trimmed with fastp as described above. SKA-fastq with a *k*-mer length of 19 and an MAF filter of 0.01 was used to measure the SNP distance between the original genome and the plasmid-altered genome. Simulated reads were re-assembled with Shovill using the parameters detailed above. The resulting genome assemblies were processed with SKA-fasta using a *k*-mer length of 37. An overview of this methodology is provided in Fig. 2b.

**Table 3.** Plasmid-modified assemblies. Where plasmids were removed from assemblies the plasmid source is marked as Not Applicable (n.a.)

Panel in Fig. 6	Species	Original genome	Original plasmid no.	Modification	Plasmid source	Modified plasmid no.
A	*E. coli*	GCF_000005845.2	0	Plus 2 plasmids	GCF_000285655.3	2
B	*E. coli*	GCF_000285655.3	2	Minus 2 plasmids	n.a.	0
C	*E. faecium*	GCF_015767695.1	0	Plus 4 plasmids	GCF_022691445.1	4
D	*E. faecium*	GCF_021398505.1	1	Minus 1 plasmid	n.a.	0
E	*E. faecium*	GCF_022691445.1	4	Minus 4 plasmids	n.a.	0
F	*K. pneumoniae*	GCF_000598005.1	5	Minus 5 plasmids	n.a.	0
G	*K. pneumoniae*	GCF_000016305.1	5	Minus 5 plasmids	n.a.	0
H	*K. pneumoniae*	GCF_000009885.1	1	Plus 5 plasmids	GCF_000598005.1	6
I	*S. aureus*	GCF_022870625.1	0	Plus 2 plasmids	GCF_022699245.1	2
J	*S. aureus*	GCF_022699245.1	2	Minus 2 plasmids	na	0

## Results

### SKA with sequencing reads: sample diversity impacts performance

**Fig. 3.**
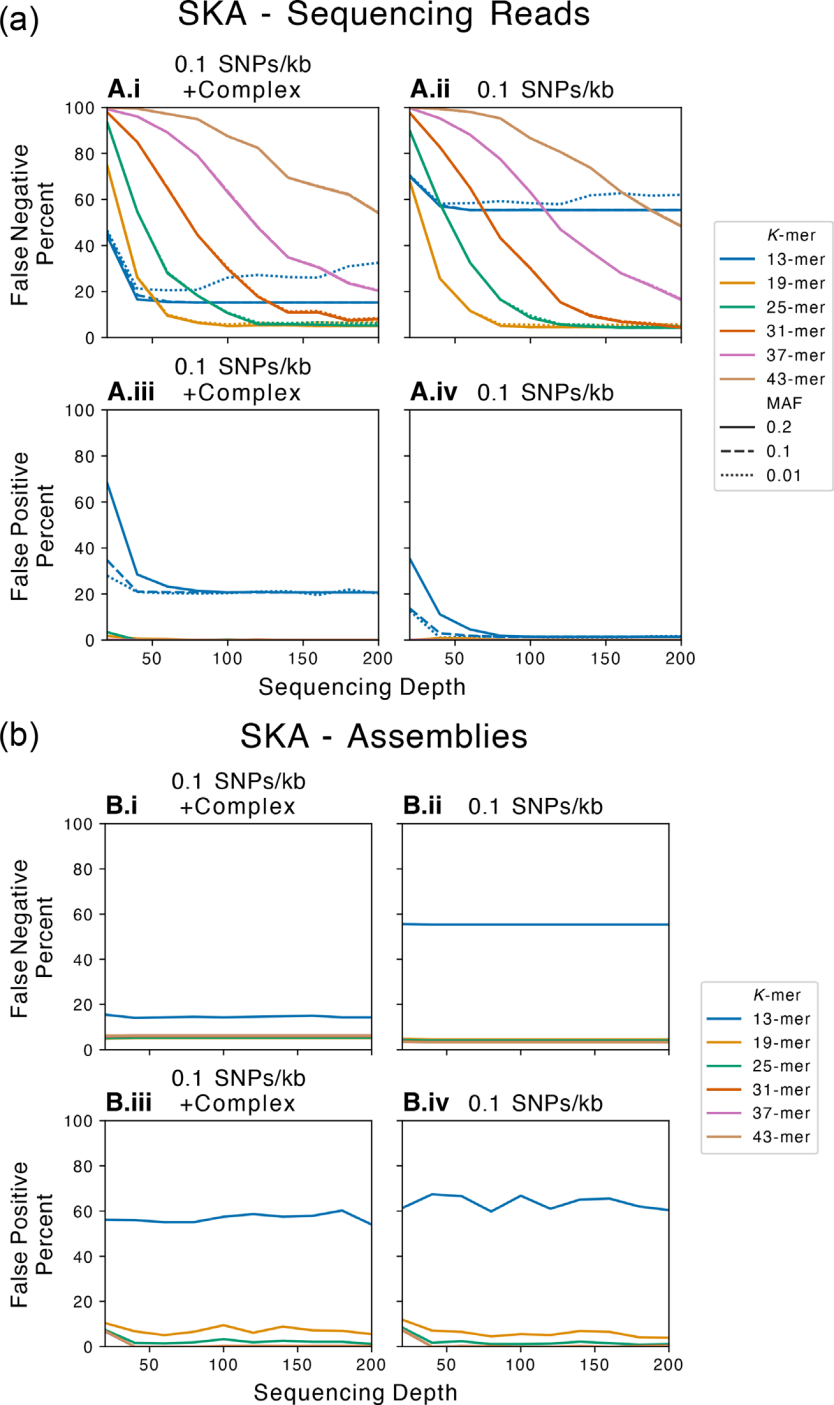
Performance of SNP detection by SKA when processing sequencing reads (**a**) or genome assemblies (**b**) from an *Escherichia coli*-simulated dataset. Percentage of SNPs (y-axis) identified by SKA classified as false negative or false positive against simulated sequencing depth (x-axis). Accuracy was assessed at a mutation rate of 0.1 SNPs per kb with complex mutations or SNP only. Lines are coloured by *k*-mer length, and line type indicates MAF filter threshold [panel (a) only]. *K*-mer lengths are inclusive of the central variable base. Default values for SKA are the 31-mer and MAF of 0.2. Except for the 13-mer (blue lines), SKA’s performance was not affected by the presence of complex mutations when processing either sequencing reads (**a**) or genome assemblies (**b**). Sequencing depth impacted SKA’s performance only when using sequencing reads (**a**), with higher depth equating to a reduced false negative percentage. Shorter *k*-mers displayed greater SNP detection at lower depths (19-mer: orange and 25-mer: green lines) compared to longer *k*-mers (43-mer: brown and 37-mer: pink lines). Sequencing depth did not affect the performance of SKA with genome assemblies (**b**). The false positive percentage remained consistently low when processing sequencing reads but was elevated in shorter *k*-mers with genome assemblies.

To assess the performance of SKA, we used a simulated dataset where we could control rates and types of variants (Fig. 2a), allowing the calculation of false negative (undetected SNPs) and false positive percentages (erroneous SNPs) (Table 2). SKA’s performance with sequencing reads degraded as the simulated mutation rate increased (mimicking increased sample diversity) (Figs S1–S12). The false negative percentage increased with higher simulated mutation rates across all *k*-mer lengths and MAF filters. A marked drop in performance was observed when the simulated mutation rate exceeded 10 SNPs per kb. As our focus was on the use of SKA for transmission detection, we focussed on SKA’s performance at a low mutation rate of 0.1 SNP per 10 kb (Fig. 3a). At this level, we observed that the lowest false negative percentage achieved by SKA was 4.3% for both 25- and 31-mers, followed by the 19-mer at 4.5% with 200-fold sequencing depth [Fig. 3a(i, ii)]. The false positive percentage was 0% for all *k*-mers except the 13-mer (1.6% at 200-fold sequencing depth) [Fig. 3a(iii, iv)]. Complex mutations (such as indels, duplications, translocations and inversions) had minimal impact on false negative percentages (19-mer: 4.5% SNP only vs. 5.1% SNP plus complex, 25-mer: 4.3% vs. 5.3%, 31-mer: 4.3% vs. 8.1%) as well as false positive percentages for all except the 13-mer (Fig. 3a). The presence of complex mutations did markedly affect the performance of the 13-mer with the false negative percentage improving whilst the false positive percentage deteriorated (Fig. 3a).

**Fig. 4.**
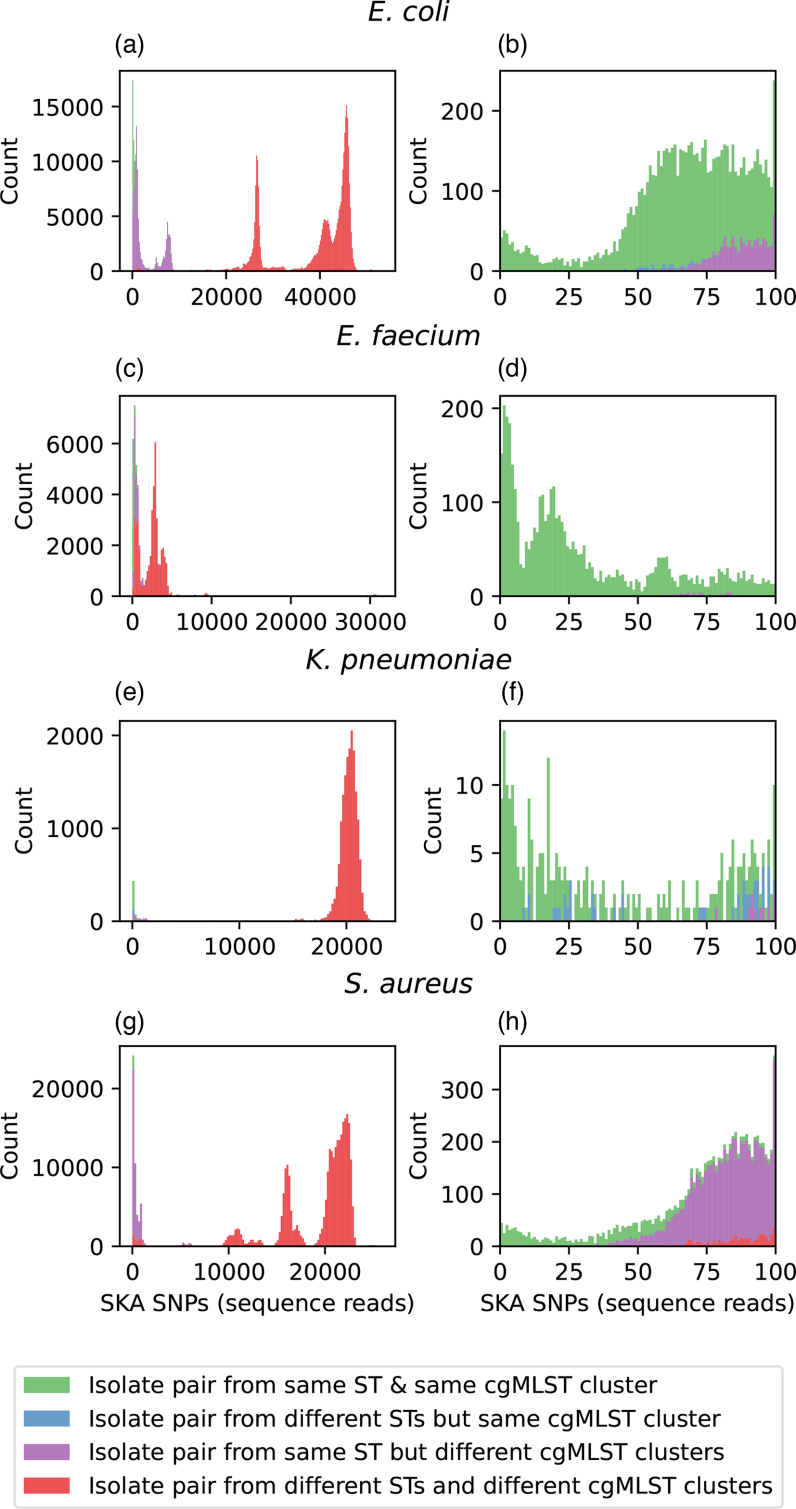
Frequency distribution of SNP distances calculated with SKA using sequencing reads from a real-world dataset as input (fastq). The SNP distance between a pair of isolates (x-axis) is plotted against the frequency with which that distance occurs (y-axis). The distributions are coloured based upon whether the pair of isolates is from the same ST and cgMLST cluster (single-linkage clustering, 25 allelic differences) (green), different ST and same cgMLST cluster (blue), same ST and different cgMLST cluster (purple) or different ST and different cgMLST cluster (red). Four bacterial species are shown [*Escherichia coli* (a, b), *Enterococcus faecium* (c, d), *K. pneumoniae* (e, f) and *S. aureus* (g, h)]. The full distributions are shown on the left [panels (a), (**c), (e) and (g**)], and the ‘zoomed in’ distribution up to a SNP distance of 100 SNPs is shown on the right [panels (b), (**d), (f) and (h**)]. Small SNP distances (indicative of closely related isolates) measured by SKA are from the same ST and cgMLST cluster, consistent with existing outbreak identification methods.

**Fig. 5.**
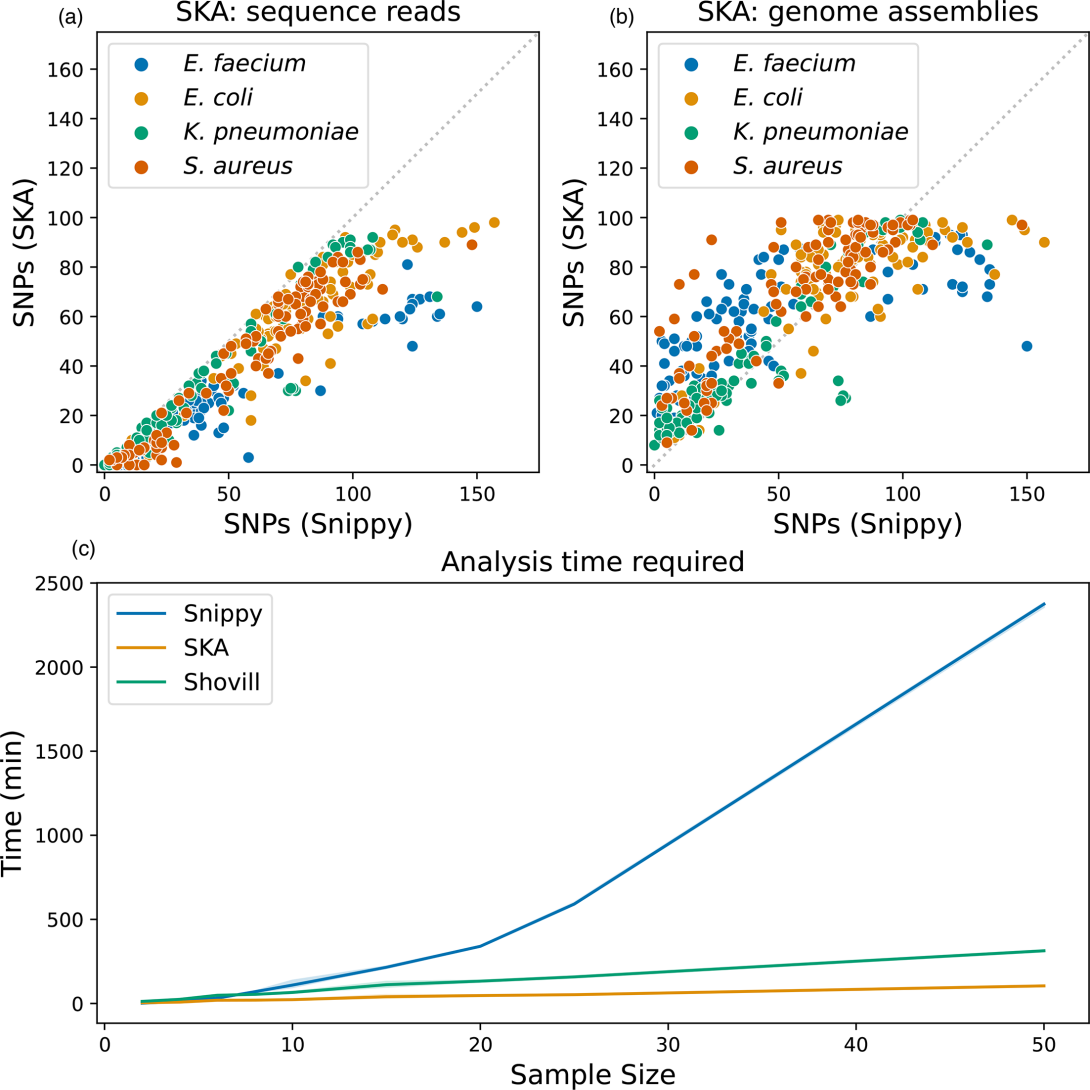
Comparison of SKA with Snippy. One hundred real-world isolates with an SKA distance of less than 100 SNPs were chosen at random for pairwise analysis with Snippy (**a, b**). SNPs detected with SKA (y-axis) using sequencing reads (**a**) or genome assemblies (**b**) are plotted against SNPs detected by Snippy (x-axis). Points are coloured by bacterial species with *Enterococcus faecium* in blue, *Escherichia coli* in gold, *K. pneumoniae* in green and *S. aureus* in red. The dotted grey line indicates equality between Snippy and SKA. (c) Amount of time taken (y-axis) by Snippy (blue line) and SKA (orange line) to perform all pairwise comparisons across different sample sizes (x-axis). Time taken by Shovill (green line) is included as *de novo* assemblies are required for pairwise Snippy analysis. Plotted lines represent the median time taken across five replicates; shaded regions indicate the minimum and maximum run times (variation in replicates was negligible).

**Fig. 6.**
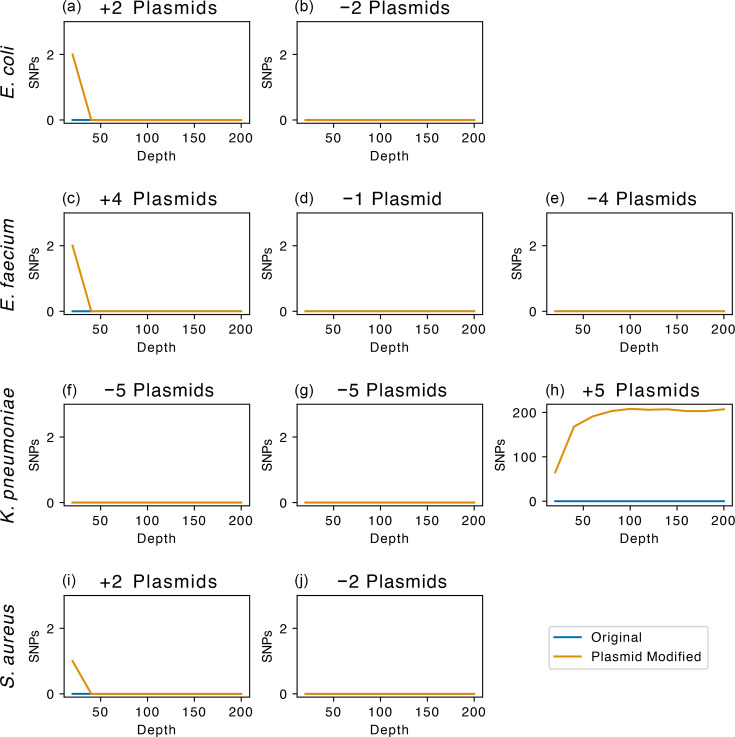
Impact of simulated plasmid movement on the detection of SNPs with SKA using sequencing reads. Number of SNPs detected with SKA (y-axis) against depth of sequencing (x-axis). In blue, the original unmodified was compared to itself as a negative control. In orange, the plasmid-modified assembly is shown. See Table 3 for details of chromosome and plasmid combinations. Note different y-axis scale for panel (h).

### SKA with sequencing reads: performance was impacted by simulated sequencing depth

The use of a simulated dataset allowed us to control additional parameters that may affect SKA’s performance, such as sequencing depth. We simulated sequencing depth ranging from 20-fold to 200-fold. Depth of sequencing differentially impacted *k*-mer performance with longer *k*-mers performing worse at lower sequencing depths (<50-fold) (Figs 3a and S1–S12). Low depth (20-fold) in combination with 31-, 37- or 43-mers failed to detect nearly all simulated SNPs [false negative: 97.6%, 99.8% and 100%, Fig. 3a(i, ii)]. Shorter *k*-mers (13, 19 and 25) were able to detect some SNPs at low depth (20-fold) with false negative percentages of 44.1%, 74.8% and 93.5%, respectively. The false negative percentage at low sequencing depth could be improved by decreasing the *k*-mer coverage cutoffs (Figs 1g and S1–S12). Using a file cutoff of 1 and a total cutoff of 2 reduced the false negative percentage to 22.6%, 31.4% and 52.0% for the 13-mer, 19-mer and 25-mer. The false positive percentage was also elevated at lower sequencing depths (13-mer: 68.3%, 19-mer: 1.8% and 25-mer: 3.4%); however, this could be rescued by decreasing the MAF filter from 0.2 to 0.01 (13-mer: 68.3% vs. 28.0%, 19-mer: 1.8% vs. 0% and 25-mer: 3.4% vs. 0%) [Fig. 3a(iii, iv)]. However, reducing the MAF filter in combination with the reduced *k*-mer coverage cutoffs (1 and 2) did not reduce the false positive per cent to 0 (13-mer: 30.8%, 19-mer: 1.0% and 25-mer: 0.93%). Reducing the MAF filter had minimal impact on the false negative percentage, regardless of sequencing depth. SKA using reads was impacted by sequencing depth, with low depth showing reduced accuracy, especially with shorter *k*-mers.

### SKA with sequencing reads: the best overall parameter combination was 19-mer, 0.01 MAF filter and default *k*-mer coverage cutoffs

The best performing parameter combination for the analysis of sequencing reads with SKA was a *k*-mer length of 19, an MAF filter of 0.01 and default coverage cutoffs. This *k*-mer length was selected as it achieved the best performance across a range of sequencing depths, whilst the MAF filter was selected to minimize the false positive rate at low sequencing depths. *K*-mer coverage cutoffs were left at default as they maintained the false positive percentage at 0, despite compromising performance at low sequencing depths (<40). Ultimately at 100-fold depth and in the presence of complex mutations, this parameter combination corresponded to an average false negative percentage of 7.1% across the four species assayed whilst the average false positive percentage was 0% (Table 3).

### SKA with genome assemblies: mutation rate impacts performance but sequencing depth does not

SKA can also process sequence data from genome assemblies. To ascertain SKA’s accuracy using assemblies, we created *de novo* genome assemblies using the simulated dataset (Table 1), enabling accurate quantification of the false negative and false positive percentages as an exploration of the impact of sequencing depth on performance. Mirroring our previous observations, the false negative percentage increased as the mutation rate was increased, whilst the inclusion of complex mutations had minimal impact (Figs. 3b and S13–S16). Focussing on a mutation rate of 0.1 SNPs per kb, SKA with genome assemblies achieved a lower false negative percentage of 3.2% (*k*-mer lengths 37 and 43) than SKA with sequencing reads (4.3%, 25- and 31-mers) (Fig. 2). However, the false positive percentage from assemblies was significantly higher than from reads, with shorter *k*-mers most affected (13-mer: 60.4% 200-fold depth and 19-mer 3.9% 200-fold) [Fig. 3b(iii, iv)]. In contrast to our results from sequencing reads, the performance of SKA with genome assemblies was not impacted by sequencing depth with equivalent performance at 20-fold (37-mer: 3.4% false negative) and 200-fold depth (37-mer: 3.2% false negative) (Fig. 3b).

### SKA with genome assemblies: overall optimum *k*-mer length was 37

Overall, the best performing *k*-mer length for analysis of genome assemblies with SKA was the 37-mer, which achieved the lowest false negative and positive percentages across the four species assayed. Using genome assemblies created from 100-fold sequencing depth with complex mutations (mimicking real-world data, rather than idealized 200-fold coverage), SKA with a 37-mer achieved an average false negative percentage of 6.5% and a false positive percentage of 0.1% across the four species tested (Table 4).

**Table 4.** The false negative and positive percentages calculated for SKA. For analysis of sequencing reads, a *k*-mer length of 19 and an MAF filter of 0.01 were used. For genome assemblies, a *k*-mer length of 37 was used. Sequencing depth was simulated at 100-fold and complex mutations were included.

Species	Sequencing read (%)		Genome assembly (%)	
	False negative	False positive	False negative	False positive
*E. coli*	5.8	0	6.2	0
*E. faecium*	14.7	0	12.2	0.4
*K. pneumoniae*	3.9	0	4.1	0
*S. aureus*	3.8	0	3.4	0
**Average**	**7.1**	**0.0**	**6.5**	**0.1**

### SNP distances computed with SKA correlate well with other typing methods

One of the principal uses for SNP distances calculated with SKA is to identify highly similar isolates with a low SNP distance, indicative of potential transmission. Existing methods for identifying potential transmission include MLST (moderate resolution, used for ‘rule-in/rule-out’ purposes) and cgMLST (commonly used to define potential transmission clusters) [5]. Hence, we compared SKA SNP distances to these other commonly used methods, using a real-world dataset [16]. SNP distances were calculated with SKA using our previously identified optimum parameters (19-mer, 0.01 MAF, file coverage cutoff of 2 and total cutoff of 4 for reads, 37-mer for genome assemblies). SKA had excellent concordance with MLST and cgMLST clusters (single-linkage clustering at 25 allelic differences). Isolate pair comparisons within the same Sequence Type (ST) and cgMLST cluster had small SNP distances, whereas isolate comparisons between different STs or different cgMLST clusters had higher SNP distances (Figs 4 and S17). Therefore, small SNP distances with SKA indicate that isolates are from the same ST and cgMLST cluster, consistent with existing outbreak detection methods.

### SKA with sequencing reads: performance on a real-world dataset

We assessed the performance of SKA in comparison to Snippy on our collection of real-world isolates. SKA was used to analyse samples using both sequencing reads and *de novo* genome assemblies with the optimum parameters determined above. For each species, 100 isolates with a SKA distance of less than 100 SNPs were randomly selected. Snippy was used to confirm the ‘true’ SNP distance between these isolate pairs (Fig. 1b). For each pair of isolates, one was randomly selected as the ‘reference’, and a *de novo* genome assembly was created, to which reads from the second ‘query’ isolate were mapped. As SKA cannot handle indels and complex mutations, these were excluded from Snippy results. Under these conditions, SKA with sequencing reads under-identified SNPs compared to Snippy, confirming our previously estimated false negative percentage of 7.1% (Fig. 5a). The performance was worst in *E. faecium* (14.7% false negative) with several outliers where Snippy identified notably more SNPs than SKA. Conversely, *K. pneumoniae* and *S. aureus* (3.9% and 3.8% false negative) performed particularly well with a good correlation between SKA and Snippy. Results from our simulated dataset demonstrated that reducing the *k*-mer coverage cutoffs could improve the false negative percentage at the cost of increased false positives. We investigated whether reducing the coverage cutoffs could improve the correlation between SKA and Snippy (Fig. S18). Adjusting the coverage cutoffs had minimal impact on the overall correlation; however, there was an increase in the number of instances where SKA identified more SNPs than Snippy (18 instances with coverage cutoffs of 1 and 2 vs. 2 instances with cutoffs of 2 and 4). In one instance, SKA identified 15 more SNPs than Snippy when using a file coverage cutoff of 1 and a total cutoff of 2. The performance when using SKA with genome assemblies was markedly worse than SKA with reads, displaying a weaker correlation to Snippy (Fig. 5b). Concerningly, there were several pairwise distances where Snippy identified less than 20 SNPs whilst SKA reported more than 20.

### SKA analysis is considerably faster than pairwise Snippy

Pairwise reference-free analyses can be performed with Snippy; however, analysis time can be considerably longer than SKA, especially with larger datasets. To assess whether pairwise comparisons with Snippy would be feasible, we recorded the time required to perform pairwise analyses with SKA and with Snippy, as well as the assembly time required to generate the *de novo* assemblies (Shovill) necessary for Snippy. We drew random samples from our real-world dataset and performed pairwise comparisons. The time taken for SKA analysis was inclusive of all steps necessary for SNP distance calculation (‘sketching’). Even with a low number of samples (10), SKA could return results faster than Snippy (22 min vs. 104 min) (Fig. 5c). With larger sample sizes (50), pairwise analysis with SKA was considerably faster than Snippy (104 min vs. 39.6 h), not including the additional time for *de novo* assembly, which for 50 samples would add 5.2 h.

### Plasmid movement does not affect SNP detection with SKA

Traditional reference genome-based read-mapping approaches are unaffected by plasmid movement as they do not form part of the core genome, but SKA includes the accessory genome in the calculation of pairwise SNPs. We investigated whether plasmid gain or loss could affect the ability of SKA to detect transmission events. Plasmid contigs were manually transferred between closed reference genome assemblies, and data were simulated from both the original and plasmid-altered assemblies (Fig. 2b). SNP detection was performed on the sequencing reads with SKA using a 19-mer and an MAF filter of 0.01. The original and plasmid-altered samples were compared to each other (Table 3, Figs 6 and S18). Across the ten assayed conditions from four species, only one condition (*K. pneumoniae*+5 plasmids) resulted in a consistent change in SNP distance (Fig. 6h). The lack of a SNP distance between the original and plasmid-altered conditions indicates that plasmid movement would not affect our ability to identify transmission events, except in exceptional circumstances. As a negative control, the original isolate was compared to itself, confirming no change in SNP detection due to read simulation (Fig. 6).

## Discussion

Here, we explore the accuracy of SKA to identify SNPs for the purpose of routine outbreak surveillance. We identify that the best parameter combination is the analysis of sequencing reads with a *k*-mer length of 19 (inclusive of central variable base), an MAF filter of 0.01 and default *k*-mer coverage cutoffs. Whilst the use of a simulated dataset allows us to establish the ground-truth value, it does not accurately reflect biology where rates of mutation may vary across the genome. This could explain why the results from our simulated dataset demonstrated that the use of SKA with genome assemblies achieved marginally lower error percentages; however, this did not hold true in our real-world dataset, where SKA on genome assemblies exhibited a high degree of variability compared to Snippy. This was of particular concern where SNP distances measured by Snippy were less than 20 SNPs whilst those reported by SKA from genome assemblies were in excess of 20 SNPs – with some over 50 SNPs. This would have dramatic implications for the detection of transmission where SNP thresholds used are typically in the range of 10–30 SNPs [25,28]. In this instance, SKA may fail to identify potential transmission links, impeding epidemiological investigations and outbreak containment. It is possible that our choice of genome assembler is also impacting our SNP results with SKA. In contrast, SKA with sequencing reads rarely over-reported SNPs in comparison to Snippy. Whilst this parameter combination did not recover 100% of the mutations (either in the simulated or real-world dataset), we concluded that this would over-identify potential transmission links, which could be subsequently verified with epidemiological data, as is recommended [16,29]. Ultimately, we recommend the use of SKA with sequencing reads, a *k*-mer length of 19, an MAF filter of 0.01 and default coverage cutoffs. Our identified optimum *k*-mer lengths for SKA are consistent with other *k*-mer optimization studies [30,32].

Implementation of novel methods for prospective pathogen surveillance in clinical or public health settings requires a robust understanding of how those methods perform and whether specific data quality considerations are necessary. Hence, we further assessed how SKA performed with varying sequencing depth and plasmid movement. We found that SKA was sensitive to sequencing depth and recommend that a minimum depth of 60-fold is required to achieve reasonable accuracy. This sequencing depth is higher than typical quality control thresholds, which commonly use 30- to 50-fold depth as a minimum [33,34]. Depth less than 60-fold could be considered acceptable with the added caveat that it may impede SNP detection, again resulting in over-identification of transmission events. Reference-genome-based approaches do not consider the accessory genome, such as plasmids, whereas SKA could theoretically be impacted by changes in the accessory genome. We determined that simulated plasmid movement does not impede SKA’s detection of SNPs between highly related genomes; therefore, plasmids do not require special consideration for our use case. This is consistent with previous publications that have identified discrete *k*-mer patterns in plasmids and chromosomes [35]. However, our simulated dataset explored a limited number of conditions; further expansion and validation with a real-world dataset is important but challenging.

Broadly, SKA performed similarly for all species investigated. SKA with *E. faecium* produced the highest error rates in the simulated dataset, as well as the greatest divergence from Snippy results. It is curious that SKA performed worse with *E. faecium* than *S. aureus* given that the two species share similar GC% and genome sizes, both of which impact the number of *k*-mers that can possibly occur in the genome. Whilst recombination and other forms of horizontal gene transfer could explain this worse performance in our real-world dataset, it does not explain the higher false negative rate observed from our simulated data where recombination events were not present. It is possible that *E. faecium* possesses a greater abundance of repetitive elements, in which SKA’s short *k*-mers cannot discriminate between, therefore impeding SNP detection. However, as discussed previously, for the purpose of identification of transmission detection, this would err on the side of caution and over-identify potential transmission links. Furthermore, there have been previous publications that have extensively explored the use of SKA for transmission detection of *E. faecium*, concluding that it was an improvement on previous methods [9,10]. Future usage of SKA for other pathogens may allow to empirically determine error rates using the methodology described here to understand whether SKA is liable to over- or under-identify transmission links. It is also important that ‘SKA-specific’ SNP thresholds are used for transmission identification, noting that these thresholds will differ from core genome thresholds. SKA-specific thresholds should be determined empirically from real-world datasets, a common approach is to examine the SNP distances between repeated isolates from the same patient (within patient SNP diversity). Empirically determining SNP thresholds in this manner will intrinsically incorporate the false negative rate of SKA.

Timely detection of transmission events is vital for tackling the spread of MDROs in clinical environments and improving patient outcomes. The use of genomics promises to increase our power to detect transmission events; however, results must be available as close to real time as possible so that infection control measures can be implemented. Core genome SNP distances are unstable, whilst pairwise Snippy comparisons are prohibitively slow for clinical implementation. Whilst SNP distances measured with SKA do not precisely match those measured by Snippy, SKA can deliver results much faster, making it more suitable for implementation. A recent major update to SKA promises even further increased speed [36]. We expect our estimates of accuracy for SKA v1.0 to be comparable with the very recently released SKA v2.0 as the underlying method of using split *k*-mers to detect SNPs has not changed. Whilst the new default *k*-mer length for SKA v2.0 is similar to our identified optimum (17-mer vs. 19-mer), other parameters are no longer relevant due to the increased functionality. Further work may be necessary to fully translate our findings to the new version of SKA. There are a multitude of other *k*-mer-based tools that offer rapid pairwise genomic comparisons, making them suitable candidates for transmission detection [31,37,39]. However, some of the metrics produced by *k*-mer-based tools can be complex for non-genomic scientists or clinicians to understand (such as the Jaccard index and Mash distance), potentially hindering their implementation into clinical practice. The concept of an SNP, however, is already relatively well understood by many in infection control, and its use for measuring genome similarity is easily translated. Together, the speed and translatability of results make SKA an attractive initial application of *k*-mer-based methods for clinical and public health microbiology. We therefore recommend the use of SKA for routine outbreak surveillance.

## supplementary material

10.1099/mgen.0.001347 Uncited Supplementary Material 1.

10.1099/mgen.0.001347 Uncited Supplementary Material 2.

## Abbreviations

cgMLST: core genome MLST
IPC: infection prevention control
MAF: minor allele frequency
MDRO: multidrug-resistant organism
MLST: multilocus sequence typing
SKA: split *k*-mer analysis
