# Supplementary material for: Rapid, reference-free identification of bacterial pathogen transmission using optimized split k-mer analysis
Source: Microb Genom. 2025 Mar 6;11(3):001347. doi: 10.1099/mgen.0.001347 (PMC11936374; doi:10.1099/mgen.0.001347)
Supplement: Uncited Supplementary Material 1. [file mgen-11-01347-s001.pdf]

Rapid, reference-free identification of bacterial pathogen transmission using optimized split *k*-mer analysis

Christopher H. Connor[1], Charlie K. Higgs[1], Kristy Horan[1,2], Jason C. Kwong[1,3], M. Lindsay Grayson[3], Benjamin P. Howden[1,2,3,4], Torsten Seemann[1,2,4], Claire L. Gorrie[1], Norelle L. Sherry[1,2,3]

1. Department of Microbiology & Immunology at the Peter Doherty Institute for Infection & Immunity, University of Melbourne, Melbourne, Victoria, Australia
2. Microbiological Diagnostic Unit (MDU) Public Health Laboratory, Department of Microbiology & Immunology at the Peter Doherty Institute for Infection & Immunity, University of Melbourne, Melbourne, Victoria, Australia
3. Department of Infectious Diseases & Immunology, Austin Health, Heidelberg, Victoria, Australia
4. Centre for Pathogen Genomics, University of Melbourne, Melbourne, Victoria, Australia

Corresponding author: Benjamin P. Howden (bhowden@unimelb.edu.au)

Supplementary Figures

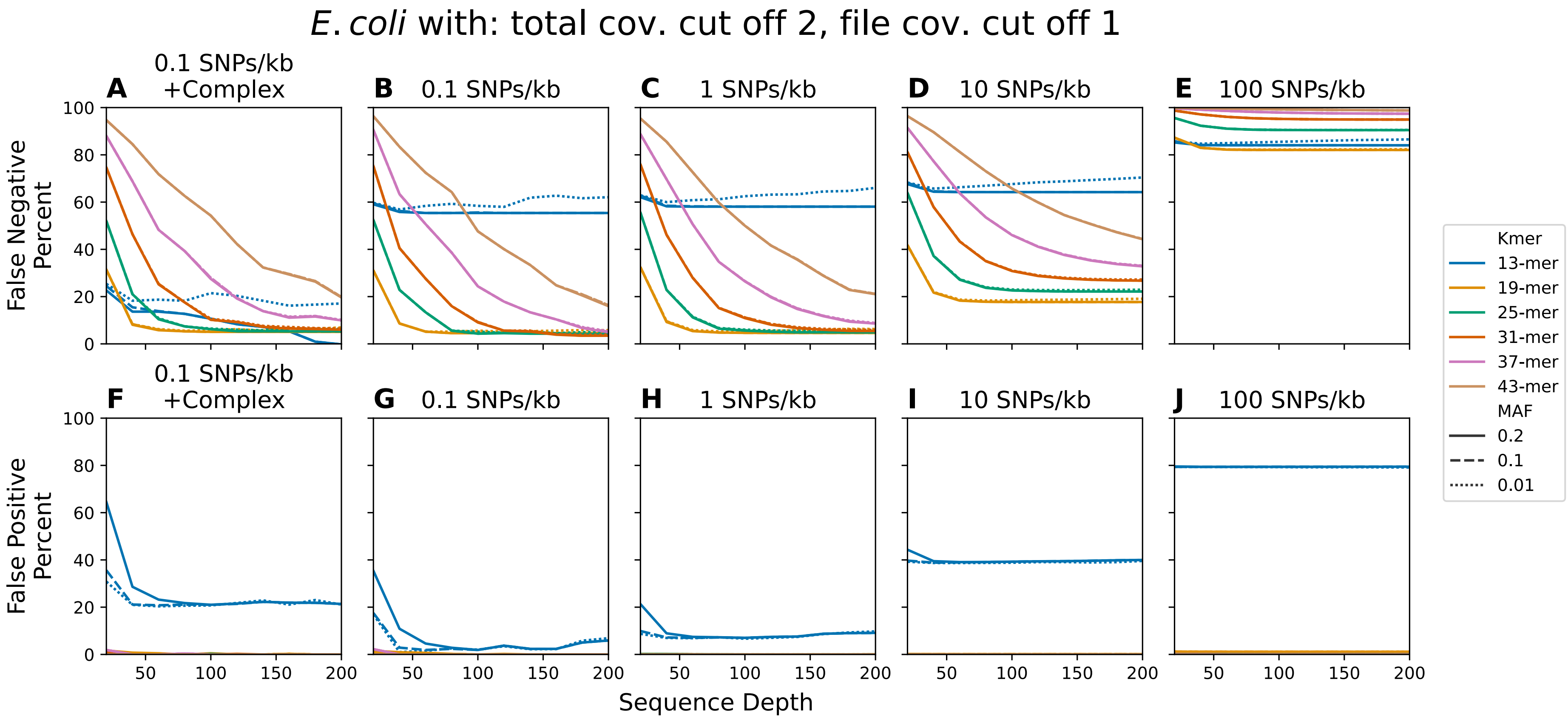

Figure S1. Performance of SNP detection by SKA when processing sequencing reads (fastq) from an *E. coli* simulated dataset with total *k*-mer coverage cutoff of 2, and a file coverage cutoff of 1. Percentage of SNPs identified by SKA classified as false negative (A-E) or false positive (F-J). Accuracy across simulated mutation rates of 0.1 SNPs per kb with complex mutations (A,F), and SNP only mutation rates of 0.1 (B, G), 1 (C, H), 10 (D, I) and 100 (E, J) SNPs per kb. Lines are coloured by *k*-mer length and line type indicates minor allele frequency filter threshold. K-mer lengths are inclusive of the central variable base. Default values for SKA are the 31-mer, MAF of 0.2, total coverage cutoff of 4 and file coverage cutoff of 2.

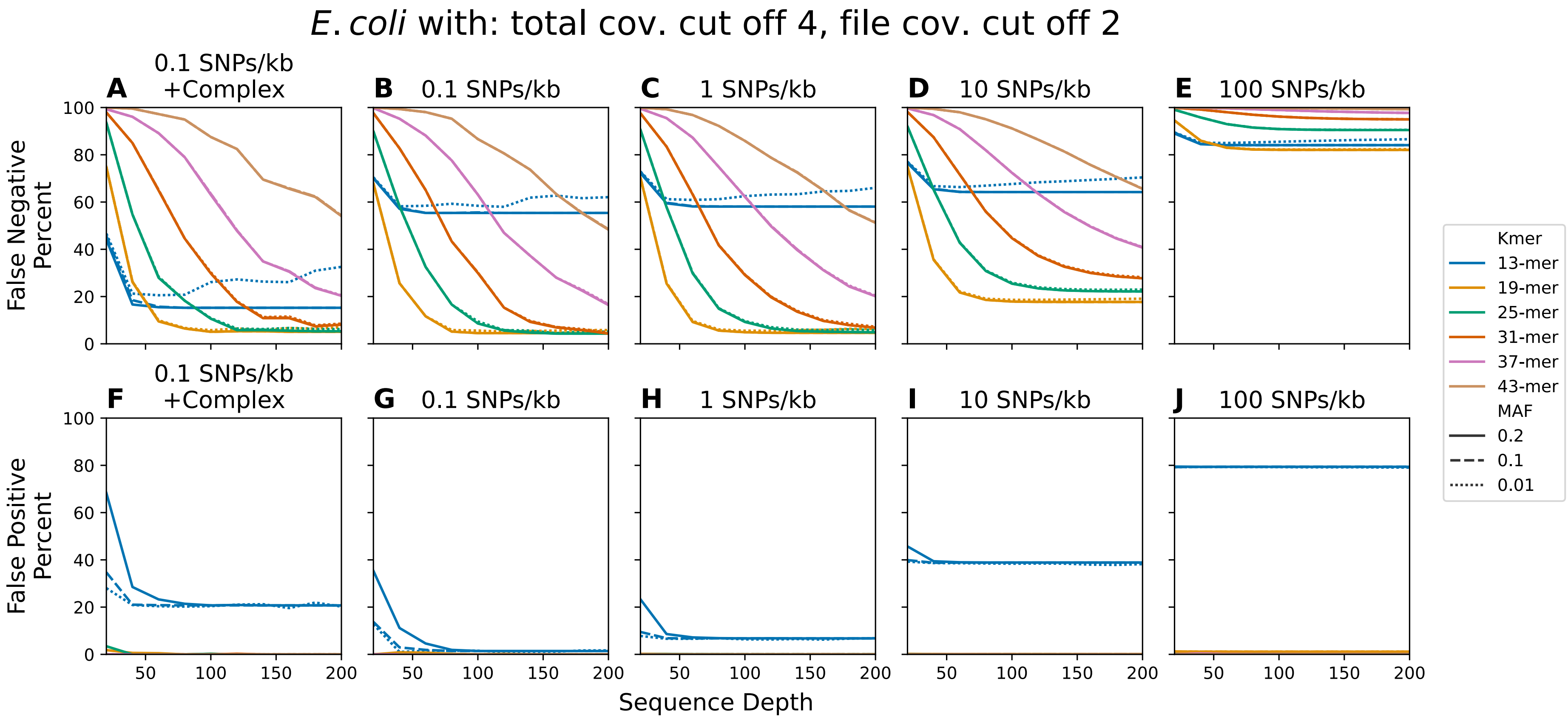

Figure S2. Performance of SNP detection by SKA when processing sequencing reads (fastq) from an *E. coli* simulated dataset with total *k*-mer coverage cutoff of 4, and a file coverage cutoff of 2. Percentage of SNPs identified by SKA classified as false negative (A-E) or false positive (F-J). Accuracy across simulated mutation rates of 0.1 SNPs per kb with complex mutations (A,F), and SNP only mutation rates of 0.1 (B, G), 1 (C, H), 10 (D, I) and 100 (E, J) SNPs per kb. Lines are coloured by *k*-mer length and line type indicates minor allele frequency filter threshold. K-mer lengths are inclusive of the central variable base. Default values for SKA are the 31-mer, MAF of 0.2, total coverage cutoff of 4 and file coverage cutoff of 2.

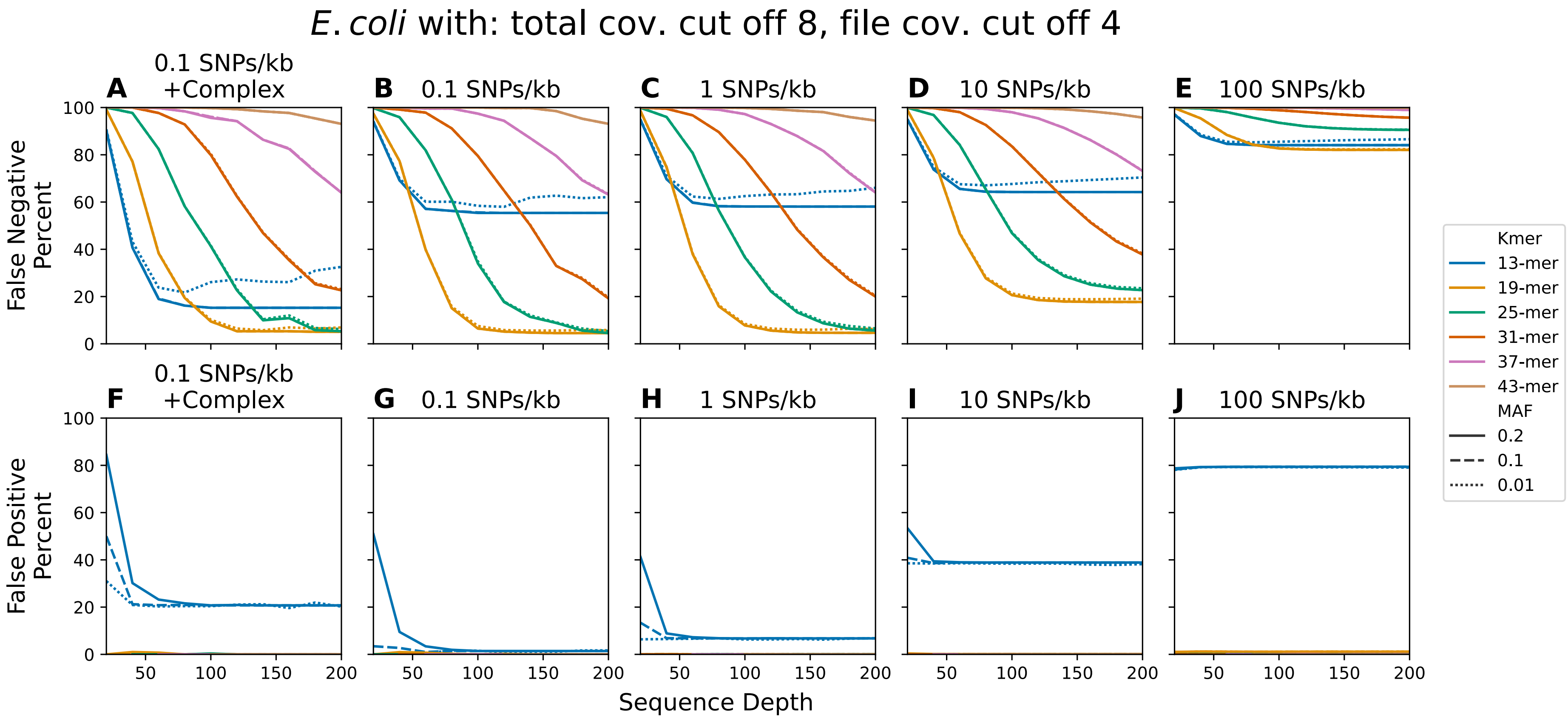

Figure S3. Performance of SNP detection by SKA when processing sequencing reads (fastq) from an *E. coli* simulated dataset with total *k*-mer coverage cutoff of 8, and a file coverage cutoff of 4. Percentage of SNPs identified by SKA classified as false negative (A-E) or false positive (F-J). Accuracy across simulated mutation rates of 0.1 SNPs per kb with complex mutations (A,F), and SNP only mutation rates of 0.1 (B, G), 1 (C, H), 10 (D, I) and 100 (E, J) SNPs per kb. Lines are coloured by *k*-mer length and line type indicates minor allele frequency filter threshold. K-mer lengths are inclusive of the central variable base. Default values for SKA are the 31-mer, MAF of 0.2, total coverage cutoff of 4 and file coverage cutoff of 2.

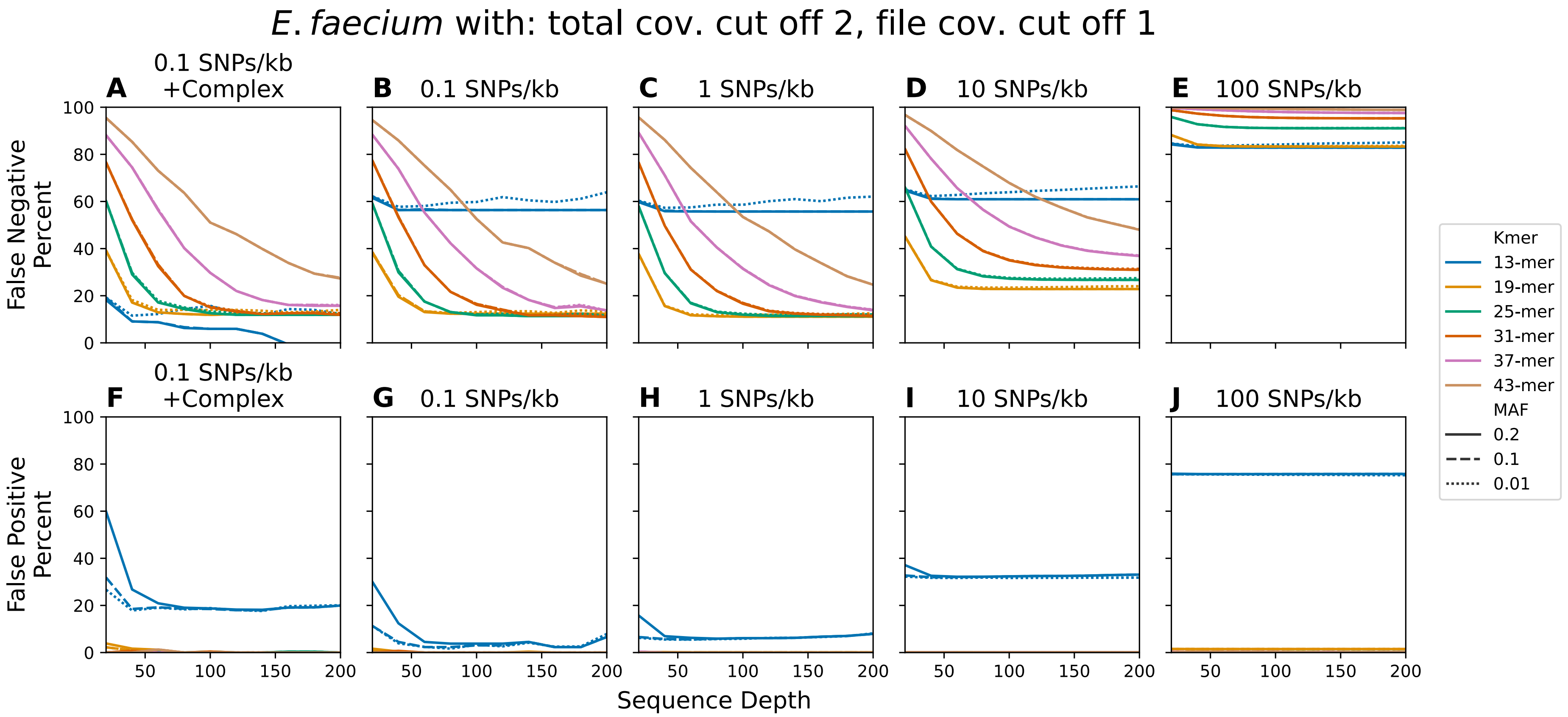

Figure S4. Performance of SNP detection by SKA when processing sequencing reads (fastq) from an *E. faecium* simulated dataset with total *k*-mer coverage cutoff of 2, and a file coverage cutoff of 1. Percentage of SNPs identified by SKA classified as false negative (A-E) or false positive (F-J). Accuracy across simulated mutation rates of 0.1 SNPs per kb with complex mutations (A,F), and SNP only mutation rates of 0.1 (B, G), 1 (C, H), 10 (D, I) and 100 (E, J) SNPs per kb. Lines are coloured by *k*-mer length and line type indicates minor allele frequency filter threshold. K-mer lengths are inclusive of the central variable base. Default values for SKA are the 31-mer, MAF of 0.2, total coverage cutoff of 4 and file coverage cutoff of 2.

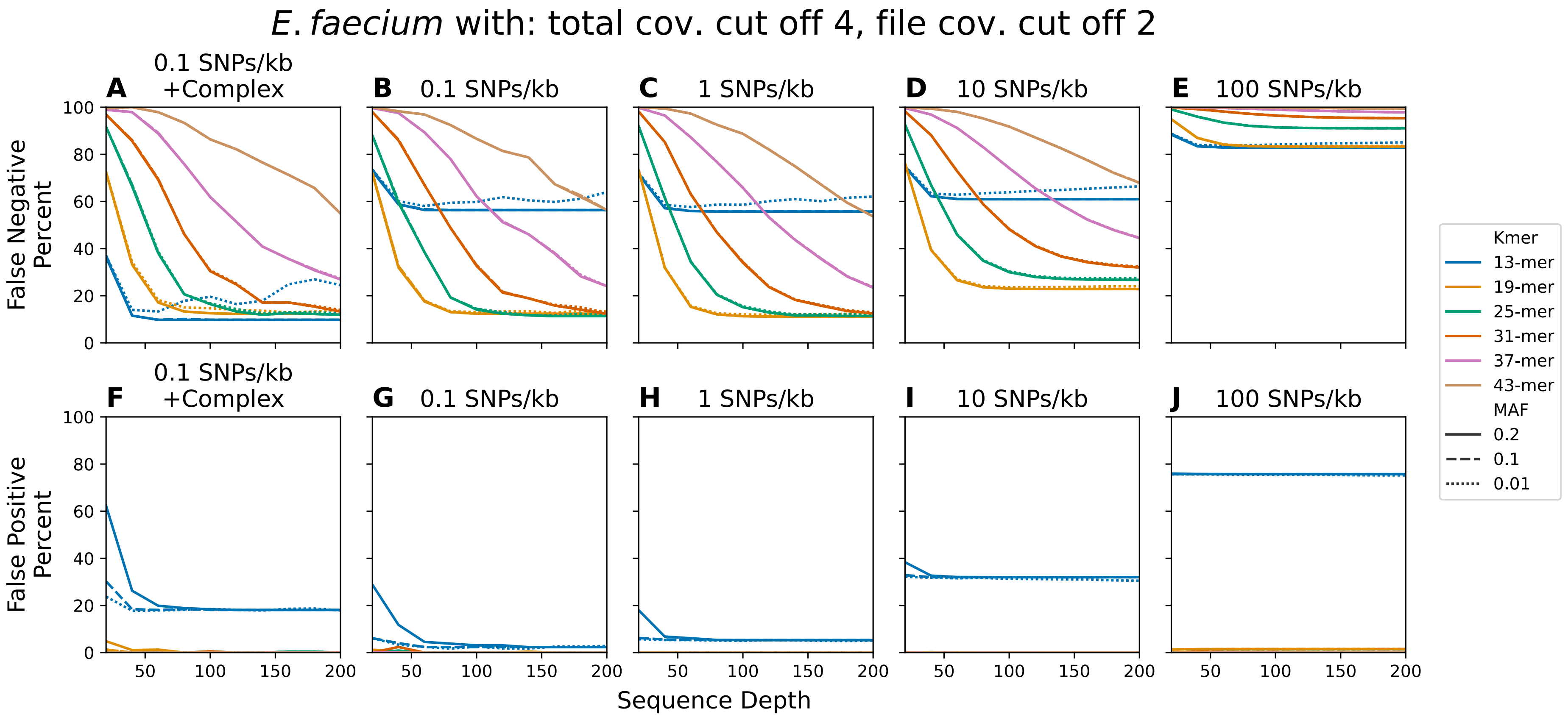

Figure S5. Performance of SNP detection by SKA when processing sequencing reads (fastq) from an *E. faecium* simulated dataset with total *k*-mer coverage cutoff of 4, and a file coverage cutoff of 2. Percentage of SNPs identified by SKA classified as false negative (A-E) or false positive (F-J). Accuracy across simulated mutation rates of 0.1 SNPs per kb with complex mutations (A,F), and SNP only mutation rates of 0.1 (B, G), 1 (C, H), 10 (D, I) and 100 (E, J) SNPs per kb. Lines are coloured by *k*-mer length and line type indicates minor allele frequency filter threshold. K-mer lengths are inclusive of the central variable base. Default values for SKA are the 31-mer, MAF of 0.2, total coverage cutoff of 4 and file coverage cutoff of 2.

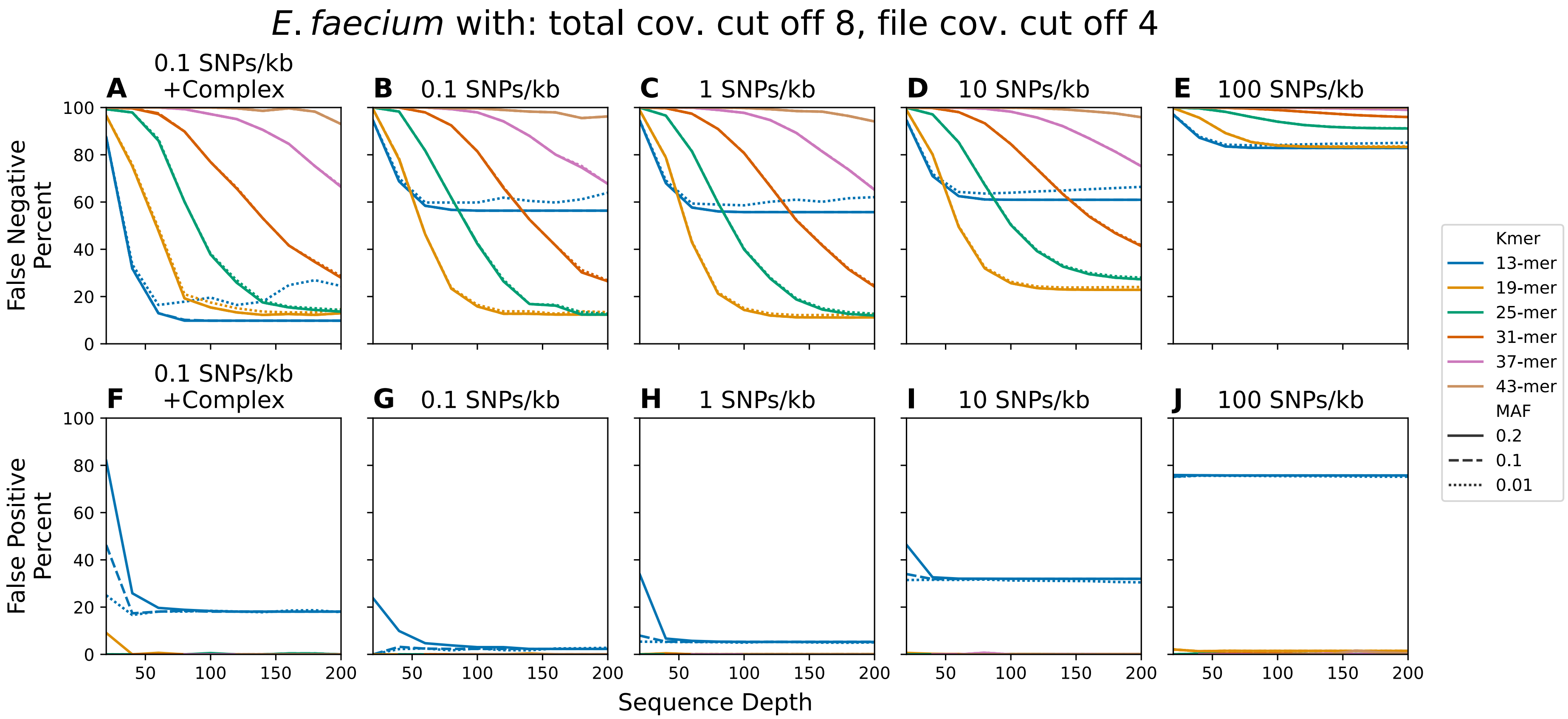

Figure S6. Performance of SNP detection by SKA when processing sequencing reads (fastq) from an *E. faecium* simulated dataset with total *k*-mer coverage cutoff of 8, and a file coverage cutoff of 4. Percentage of SNPs identified by SKA classified as false negative (A-E) or false positive (F-J). Accuracy across simulated mutation rates of 0.1 SNPs per kb with complex mutations (A,F), and SNP only mutation rates of 0.1 (B, G), 1 (C, H), 10 (D, I) and 100 (E, J) SNPs per kb. Lines are coloured by *k*-mer length and line type indicates minor allele frequency filter threshold. K-mer lengths are inclusive of the central variable base. Default values for SKA are the 31-mer, MAF of 0.2, total coverage cutoff of 4 and file coverage cutoff of 2.

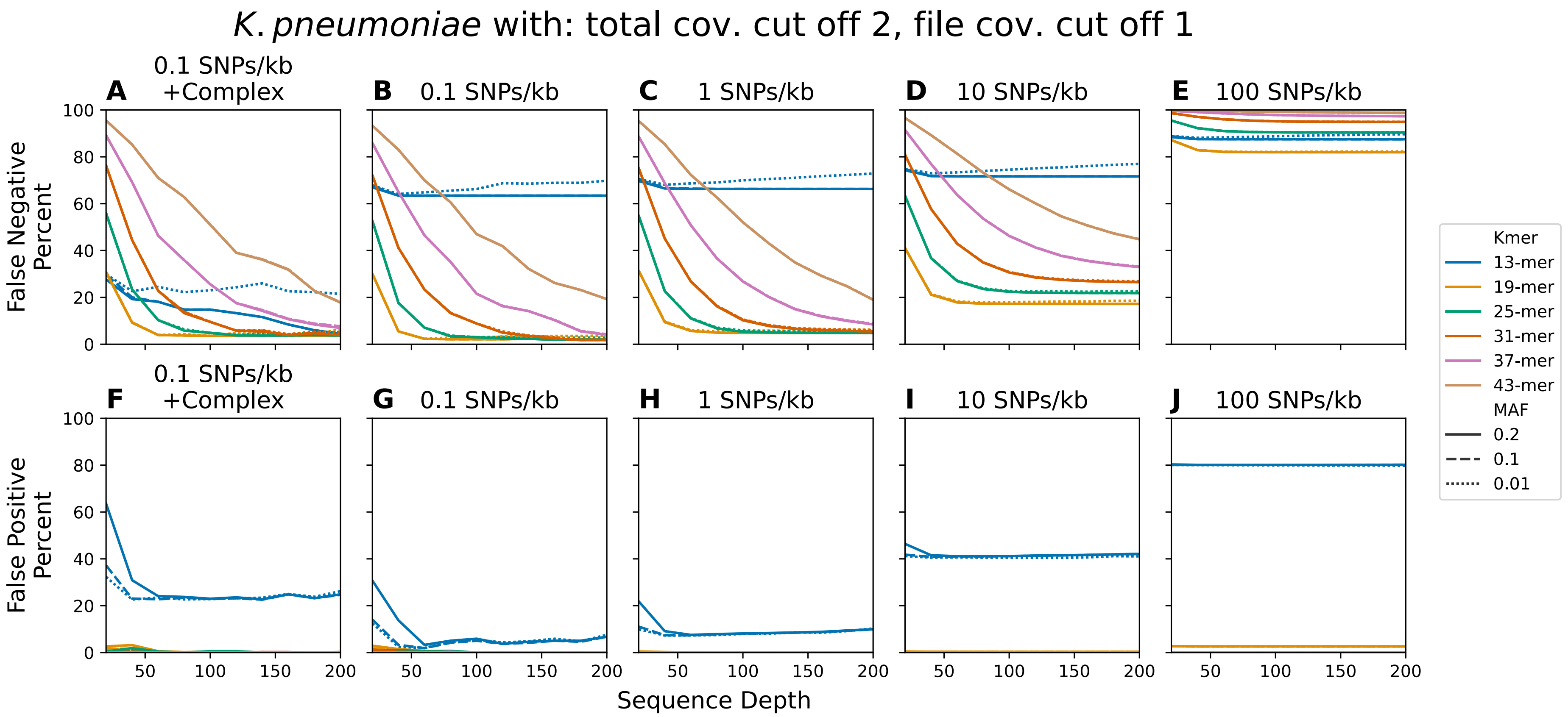

Figure S7. Performance of SNP detection by SKA when processing sequencing reads (fastq) from an *K. pneumoniae* simulated dataset with total *k*-mer coverage cutoff of 2, and a file coverage cutoff of 1. Percentage of SNPs identified by SKA classified as false negative (A-E) or false positive (F-J). Accuracy across simulated mutation rates of 0.1 SNPs per kb with complex mutations (A,F), and SNP only mutation rates of 0.1 (B, G), 1 (C, H), 10 (D, I) and 100 (E, J) SNPs per kb. Lines are coloured by *k*-mer length and line type indicates minor allele frequency filter threshold. K-mer lengths are inclusive of the central variable base. Default values for SKA are the 31-mer, MAF of 0.2, total coverage cutoff of 4 and file coverage cutoff of 2.

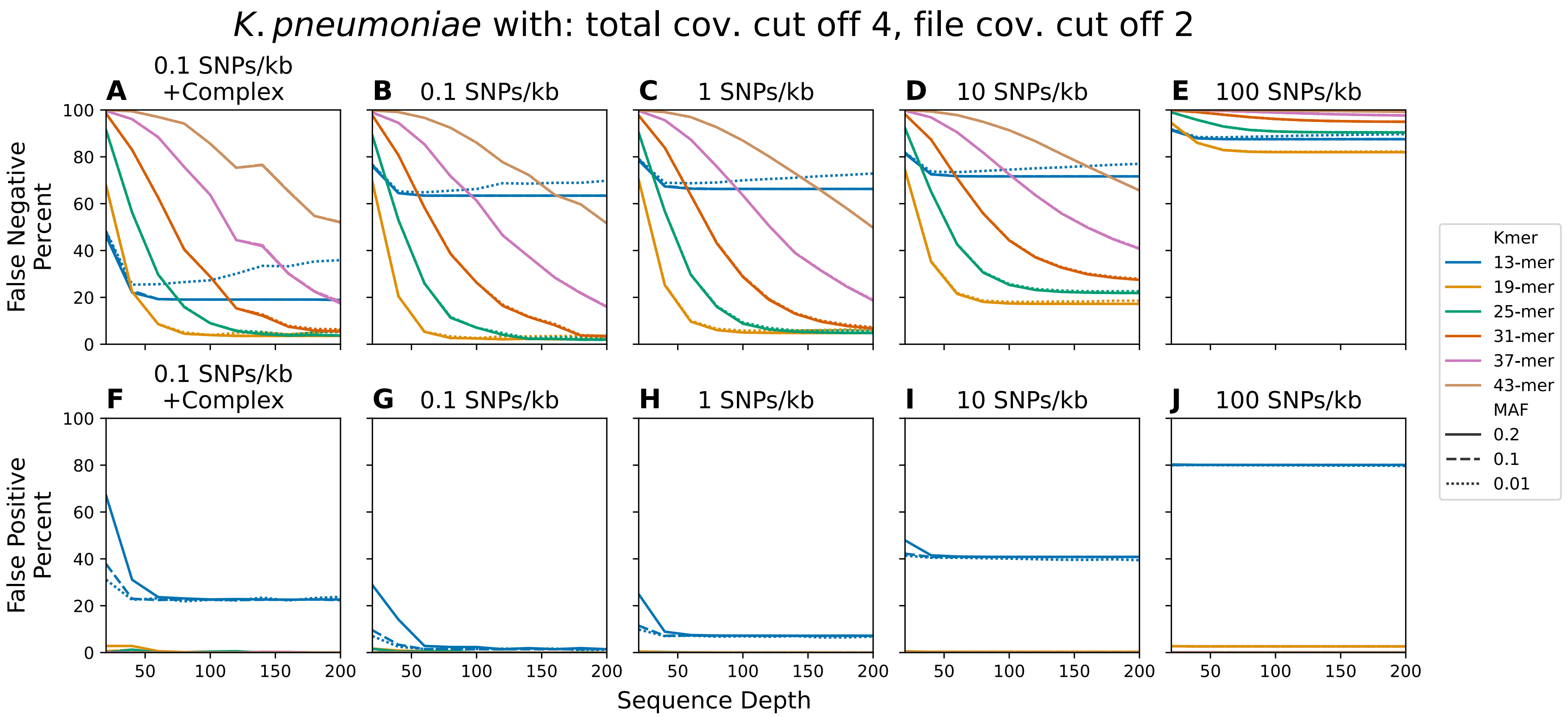

Figure S8. Performance of SNP detection by SKA when processing sequencing reads (fastq) from an *K. pneumoniae* simulated dataset with total *k*-mer coverage cutoff of 4, and a file coverage cutoff of 2. Percentage of SNPs identified by SKA classified as false negative (A-E) or false positive (F-J). Accuracy across simulated mutation rates of 0.1 SNPs per kb with complex mutations (A,F), and SNP only mutation rates of 0.1 (B, G), 1 (C, H), 10 (D, I) and 100 (E, J) SNPs per kb. Lines are coloured by *k*-mer length and line type indicates minor allele frequency filter threshold. K-mer lengths are inclusive of the central variable base. Default values for SKA are the 31-mer, MAF of 0.2, total coverage cutoff of 4 and file coverage cutoff of 2.

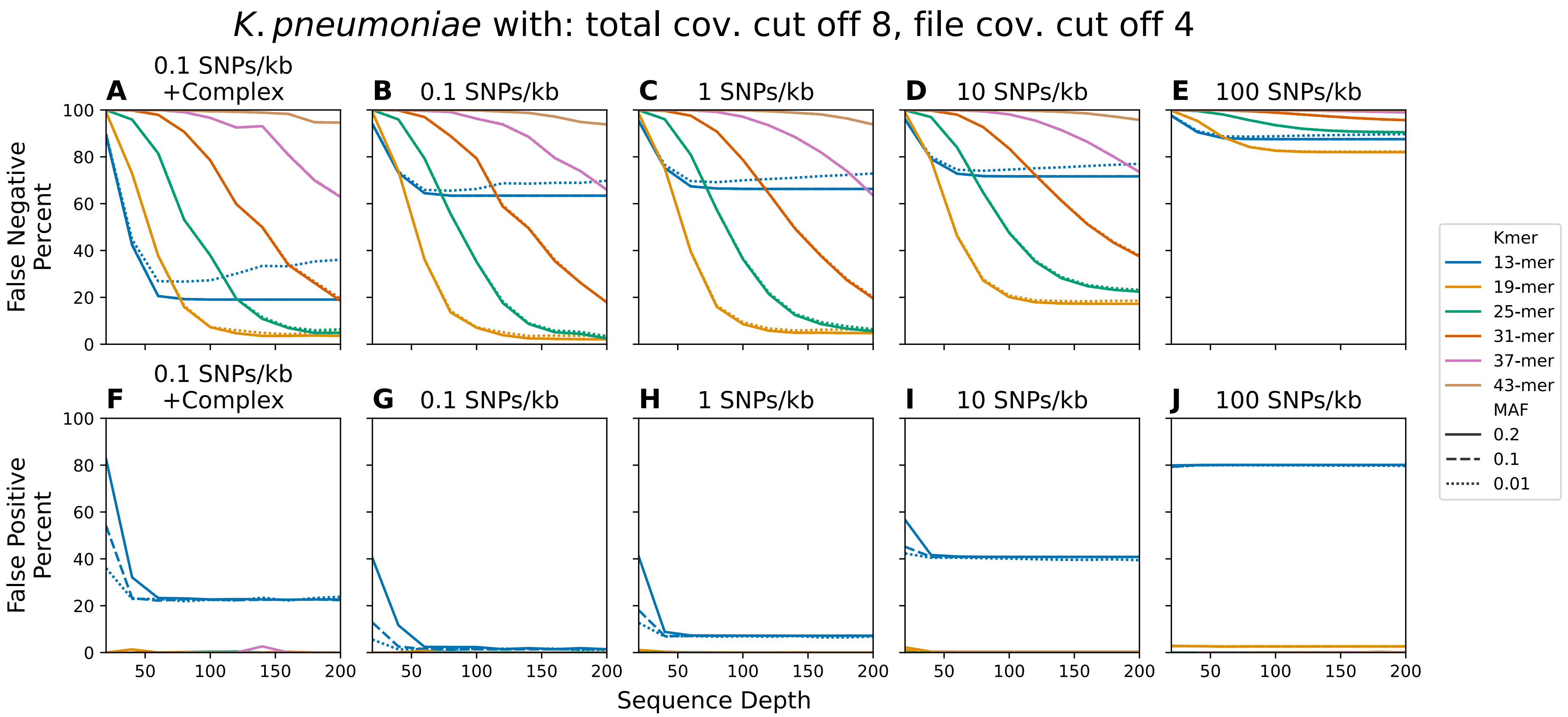

Figure S9. Performance of SNP detection by SKA when processing sequencing reads (fastq) from an *K. pneumoniae* simulated dataset with total *k*-mer coverage cutoff of 8, and a file coverage cutoff of 4. Percentage of SNPs identified by SKA classified as false negative (A-E) or false positive (F-J). Accuracy across simulated mutation rates of 0.1 SNPs per kb with complex mutations (A,F), and SNP only mutation rates of 0.1 (B, G), 1 (C, H), 10 (D, I) and 100 (E, J) SNPs per kb. Lines are coloured by *k*-mer length and line type indicates minor allele frequency filter threshold. K-mer lengths are inclusive of the central variable base. Default values for SKA are the 31-mer, MAF of 0.2, total coverage cutoff of 4 and file coverage cutoff of 2.

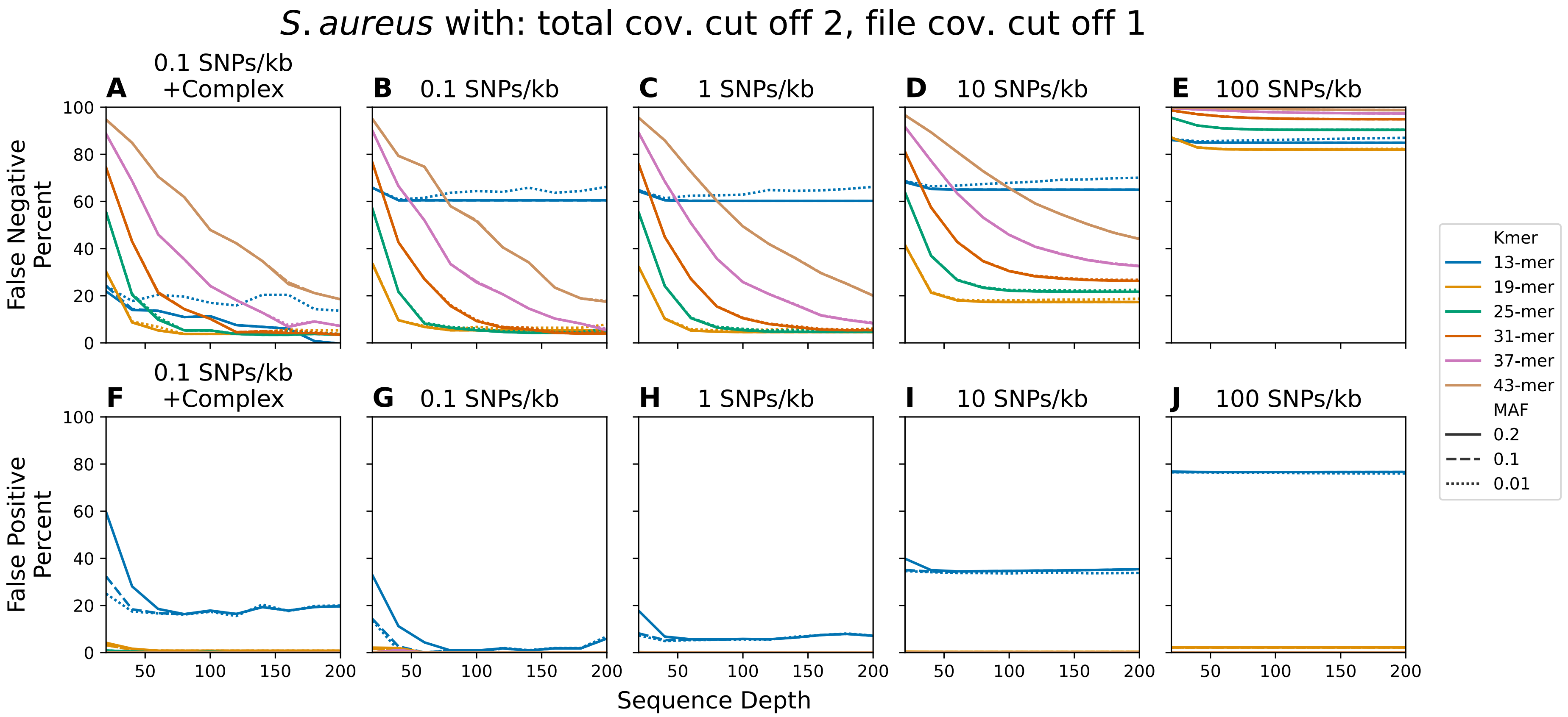

Figure S10. Performance of SNP detection by SKA when processing sequencing reads (fastq) from an *S. aureus* simulated dataset with total *k*-mer coverage cutoff of 2, and a file coverage cutoff of 1. Percentage of SNPs identified by SKA classified as false negative (A-E) or false positive (F-J). Accuracy across simulated mutation rates of 0.1 SNPs per kb with complex mutations (A,F), and SNP only mutation rates of 0.1 (B, G), 1 (C, H), 10 (D, I) and 100 (E, J) SNPs per kb. Lines are coloured by *k*-mer length and line type indicates minor allele frequency filter threshold. K-mer lengths are inclusive of the central variable base. Default values for SKA are the 31-mer, MAF of 0.2, total coverage cutoff of 4 and file coverage cutoff of 2.

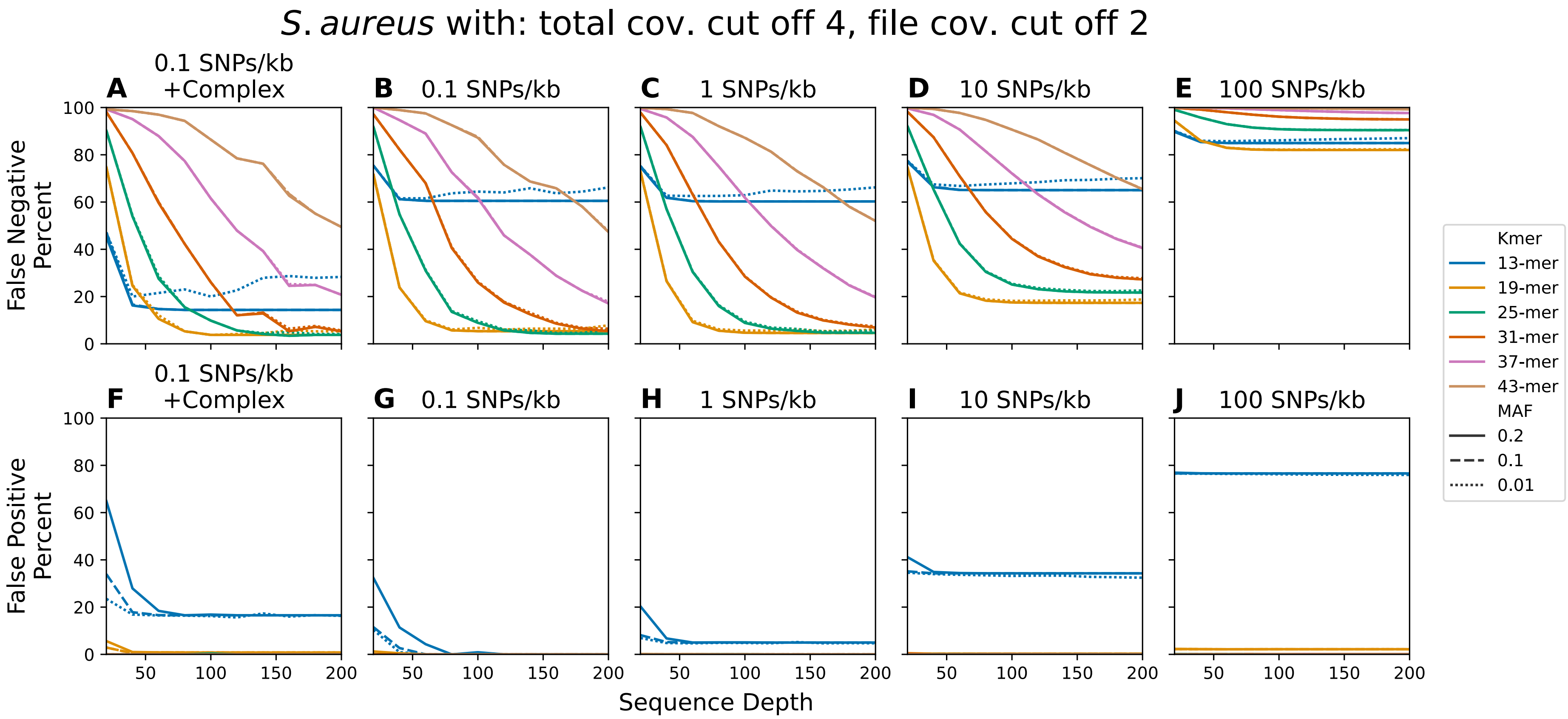

Figure S11. Performance of SNP detection by SKA when processing sequencing reads (fastq) from an *S. aureus* simulated dataset with total *k*-mer coverage cutoff of 4, and a file coverage cutoff of 2. Percentage of SNPs identified by SKA classified as false negative (A-E) or false positive (F-J). Accuracy across simulated mutation rates of 0.1 SNPs per kb with complex mutations (A,F), and SNP only mutation rates of 0.1 (B, G), 1 (C, H), 10 (D, I) and 100 (E, J) SNPs per kb. Lines are coloured by *k*-mer length and line type indicates minor allele frequency filter threshold. K-mer lengths are inclusive of the central variable base. Default values for SKA are the 31-mer, MAF of 0.2, total coverage cutoff of 4 and file coverage cutoff of 2.

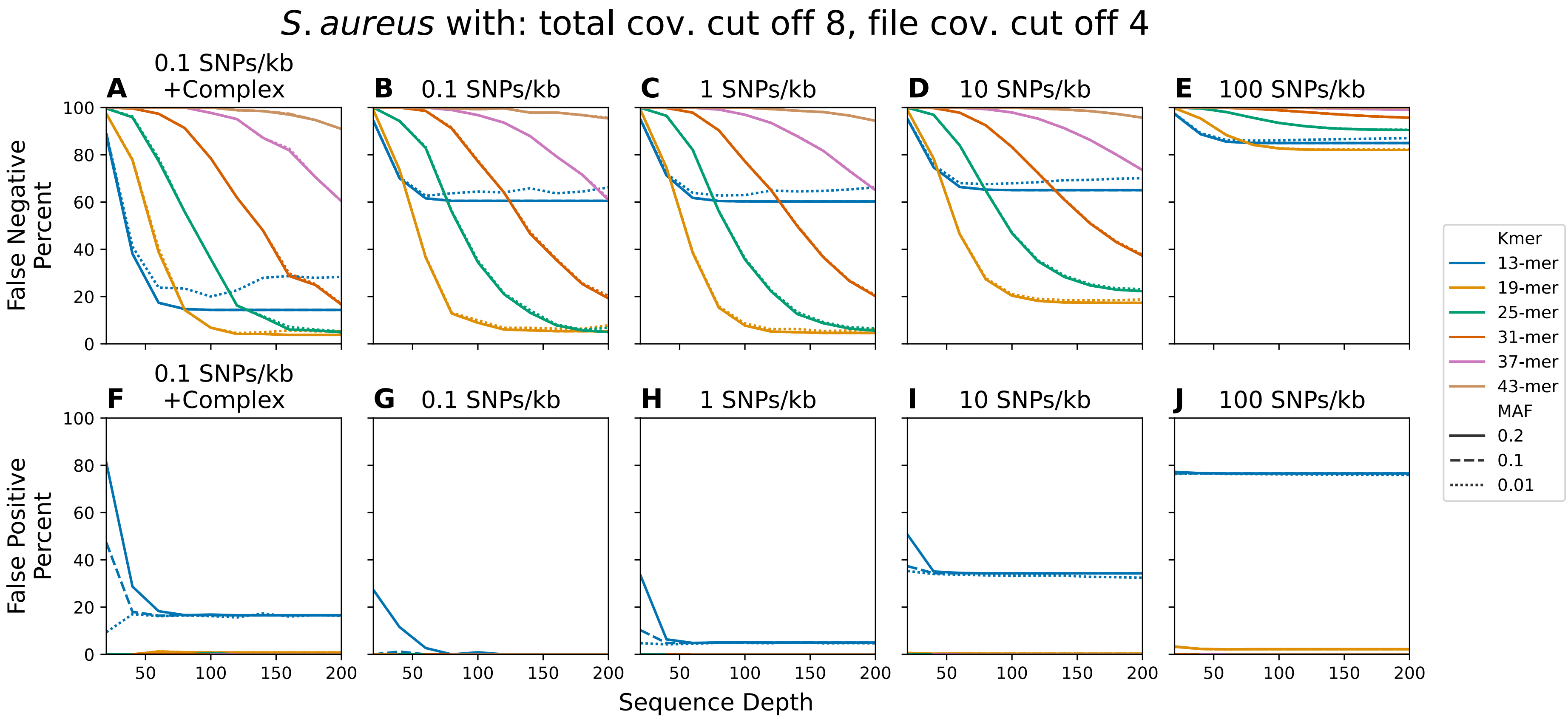

Figure S12. Performance of SNP detection by SKA when processing sequencing reads (fastq) from an *S. aureus* simulated dataset with total *k*-mer coverage cutoff of 8, and a file coverage cutoff of 4. Percentage of SNPs identified by SKA classified as false negative (A-E) or false positive (F-J). Accuracy across simulated mutation rates of 0.1 SNPs per kb with complex mutations (A,F), and SNP only mutation rates of 0.1 (B, G), 1 (C, H), 10 (D, I) and 100 (E, J) SNPs per kb. Lines are coloured by *k*-mer length and line type indicates minor allele frequency filter threshold. K-mer lengths are inclusive of the central variable base. Default values for SKA are the 31-mer, MAF of 0.2, total coverage cutoff of 4 and file coverage cutoff of 2.

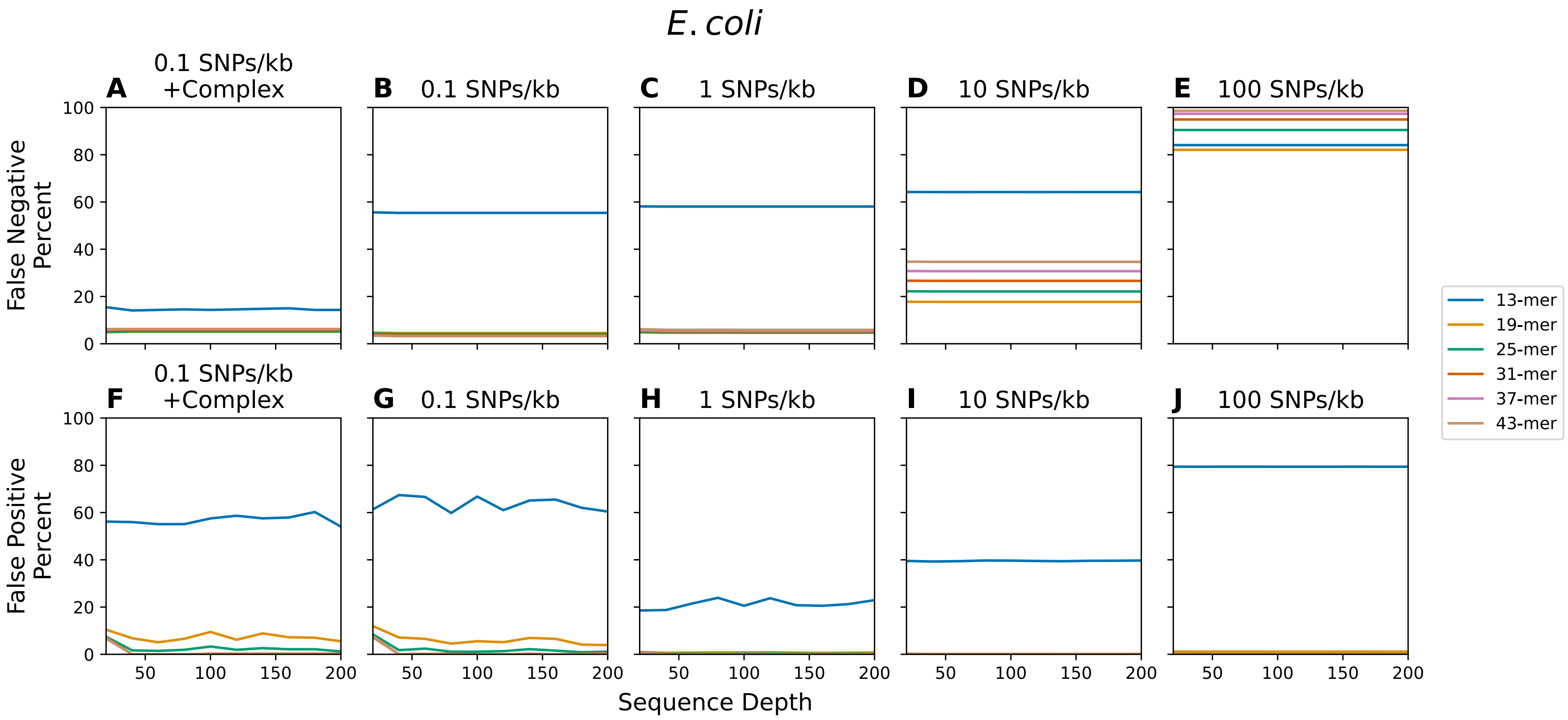

Figure S13. Performance of SNP detection by SKA when processing genome assemblies (fasta) from an *E. coli* simulated dataset. Percentage of SNPs identified by SKA classified as false negative (A-E) or false positive (F-J). Accuracy across simulated mutation rates of 0.1 SNPs per kb with complex mutations (A,F), and SNP only mutation rates of 0.1 (B, G), 1 (C, H), 10 (D, I) and 100 (E, J) SNPs per kb. Lines are coloured by *k*-mer length. K-mer lengths are inclusive of the central variable base. Default values for SKA are the 31-mer and MAF of 0.2.

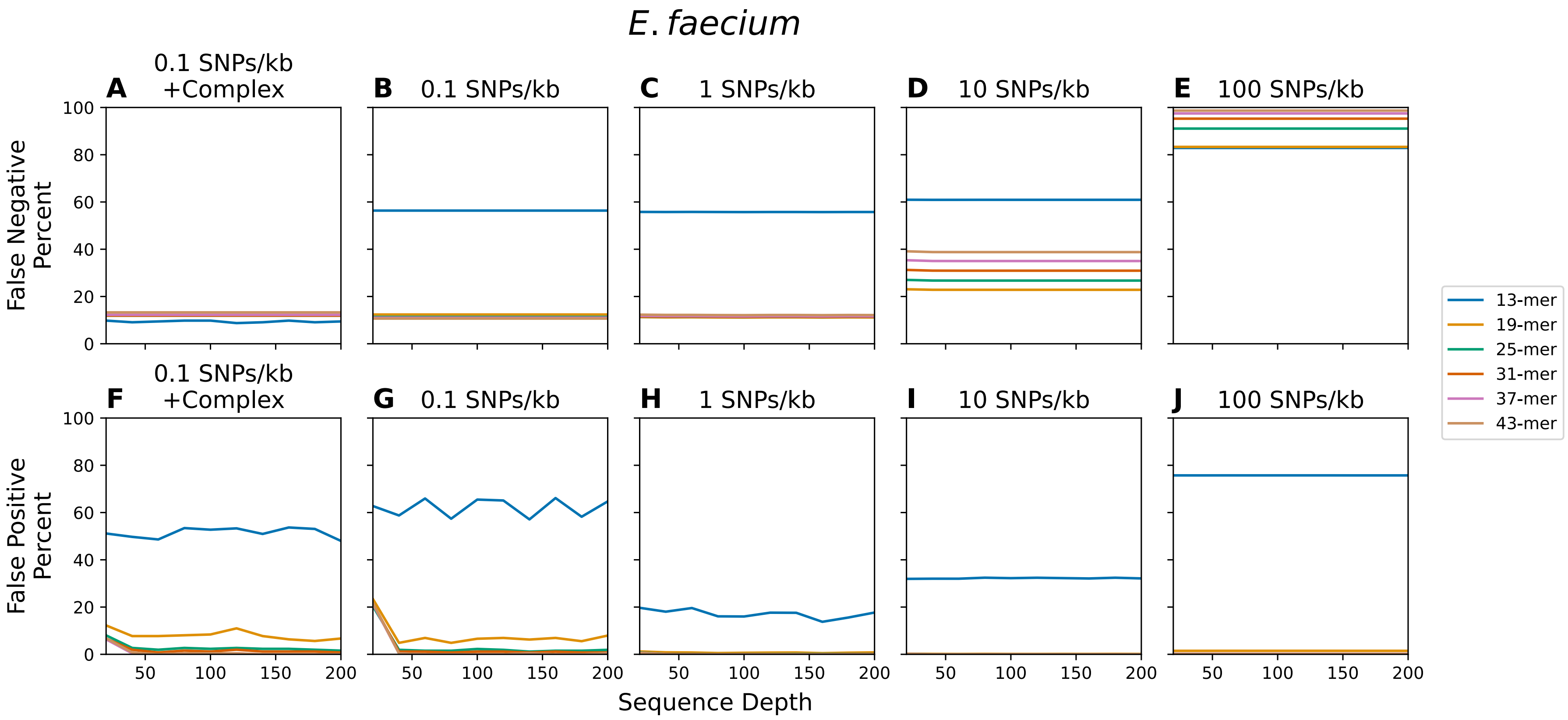

Figure S14. Performance of SNP detection by SKA when processing genome assemblies (fasta) from an *E. faecium* simulated dataset. Percentage of SNPs identified by SKA classified as false negative (A-E) or false positive (F-J). Accuracy across simulated mutation rates of 0.1 SNPs per kb with complex mutations (A,F), and SNP only mutation rates of 0.1 (B, G), 1 (C, H), 10 (D, I) and 100 (E, J) SNPs per kb. Lines are coloured by *k*-mer length. K-mer lengths are inclusive of the central variable base. Default values for SKA are the 31-mer and MAF of 0.2.

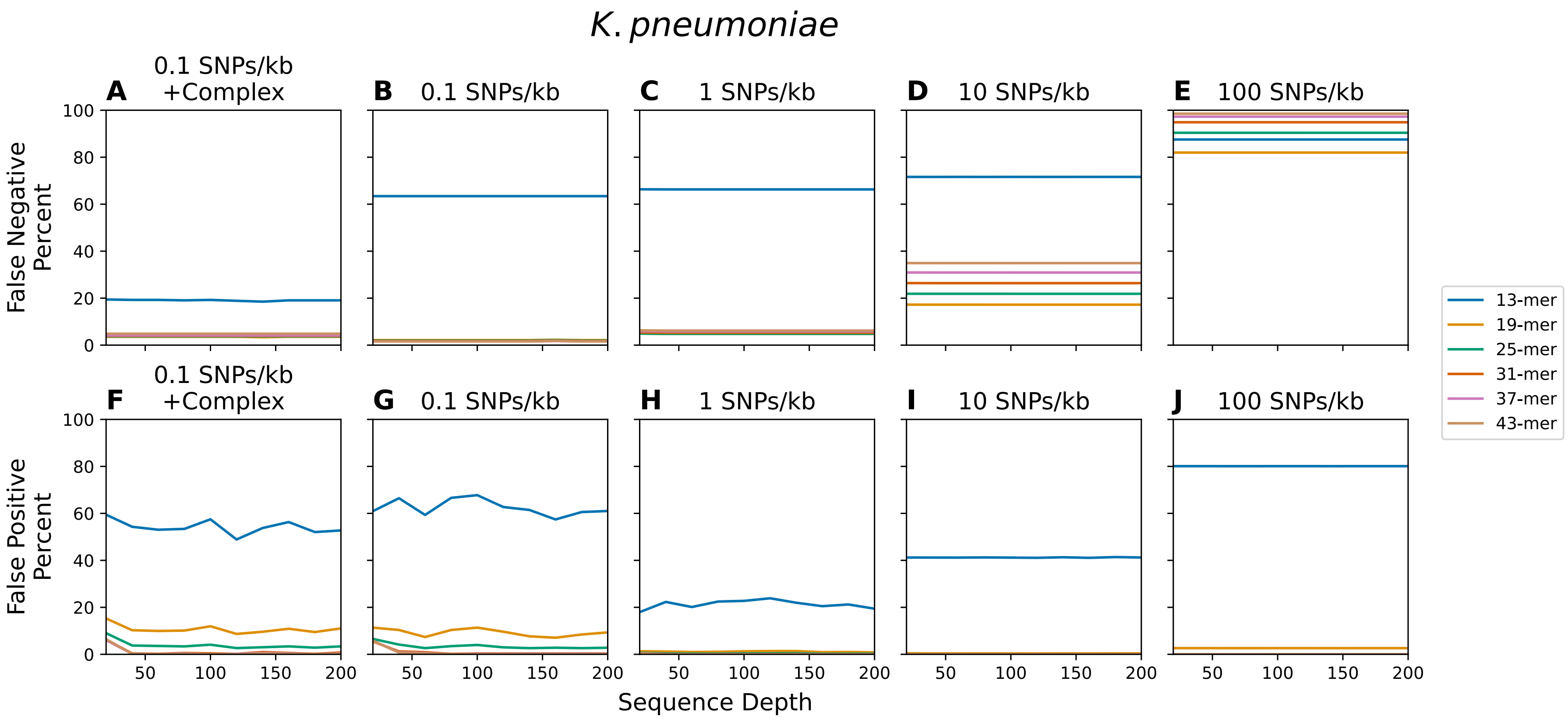

Figure S15. Performance of SNP detection by SKA when processing genome assemblies (fasta) from an *K. pneumoniae* simulated dataset. Percentage of SNPs identified by SKA classified as false negative (A-E) or false positive (F-J). Accuracy across simulated mutation rates of 0.1 SNPs per kb with complex mutations (A,F), and SNP only mutation rates of 0.1 (B, G), 1 (C, H), 10 (D, I) and 100 (E, J) SNPs per kb. Lines are coloured by *k*-mer length. K-mer lengths are inclusive of the central variable base. Default values for SKA are the 31-mer and MAF of 0.2.

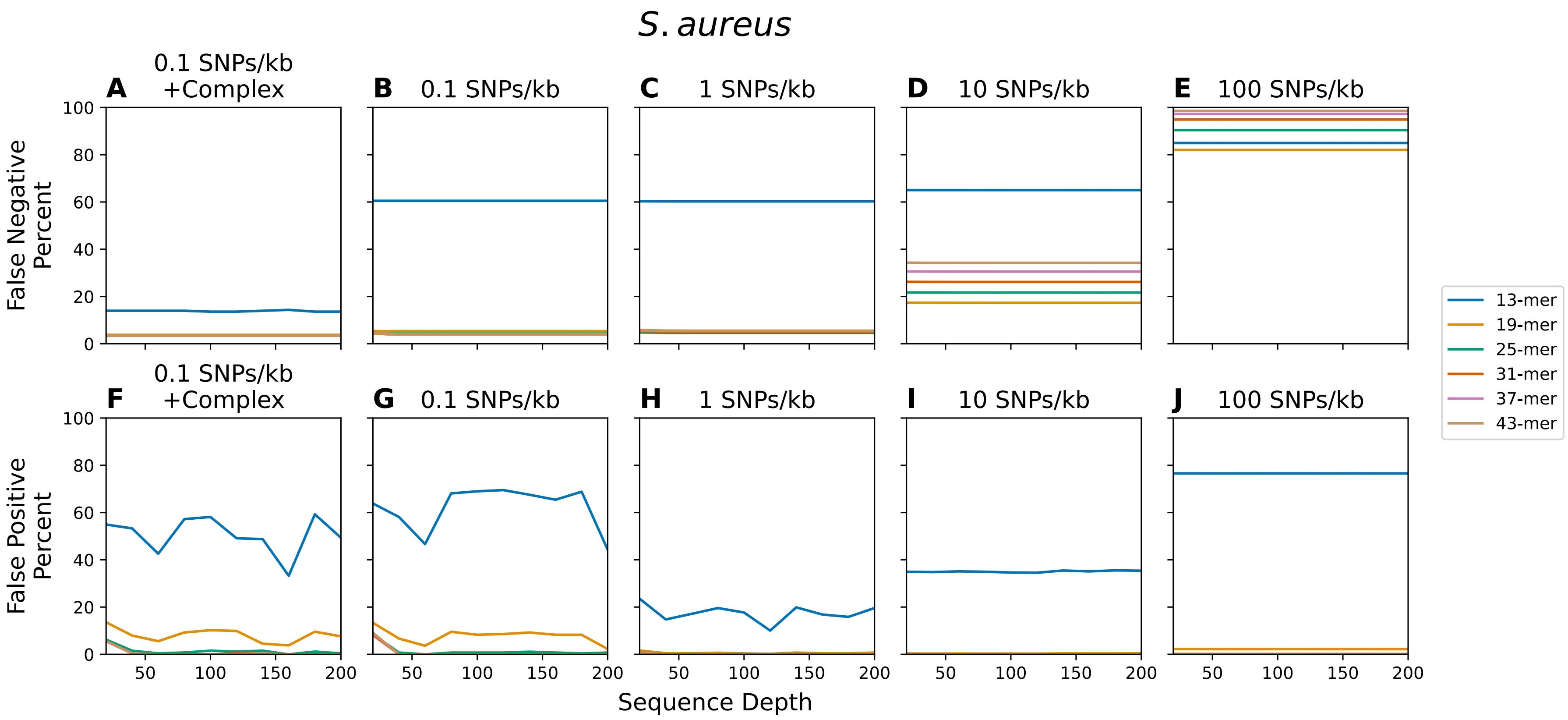

Figure S16. Performance of SNP detection by SKA when processing genome assemblies (fasta) from an *S. aureus* simulated dataset. Percentage of SNPs identified by SKA classified as false negative (A-E) or false positive (F-J). Accuracy across simulated mutation rates of 0.1 SNPs per kb with complex mutations (A,F), and SNP only mutation rates of 0.1 (B, G), 1 (C, H), 10 (D, I) and 100 (E, J) SNPs per kb. Lines are coloured by *k*-mer length. K-mer lengths are inclusive of the central variable base. Default values for SKA are the 31-mer and MAF of 0.2.

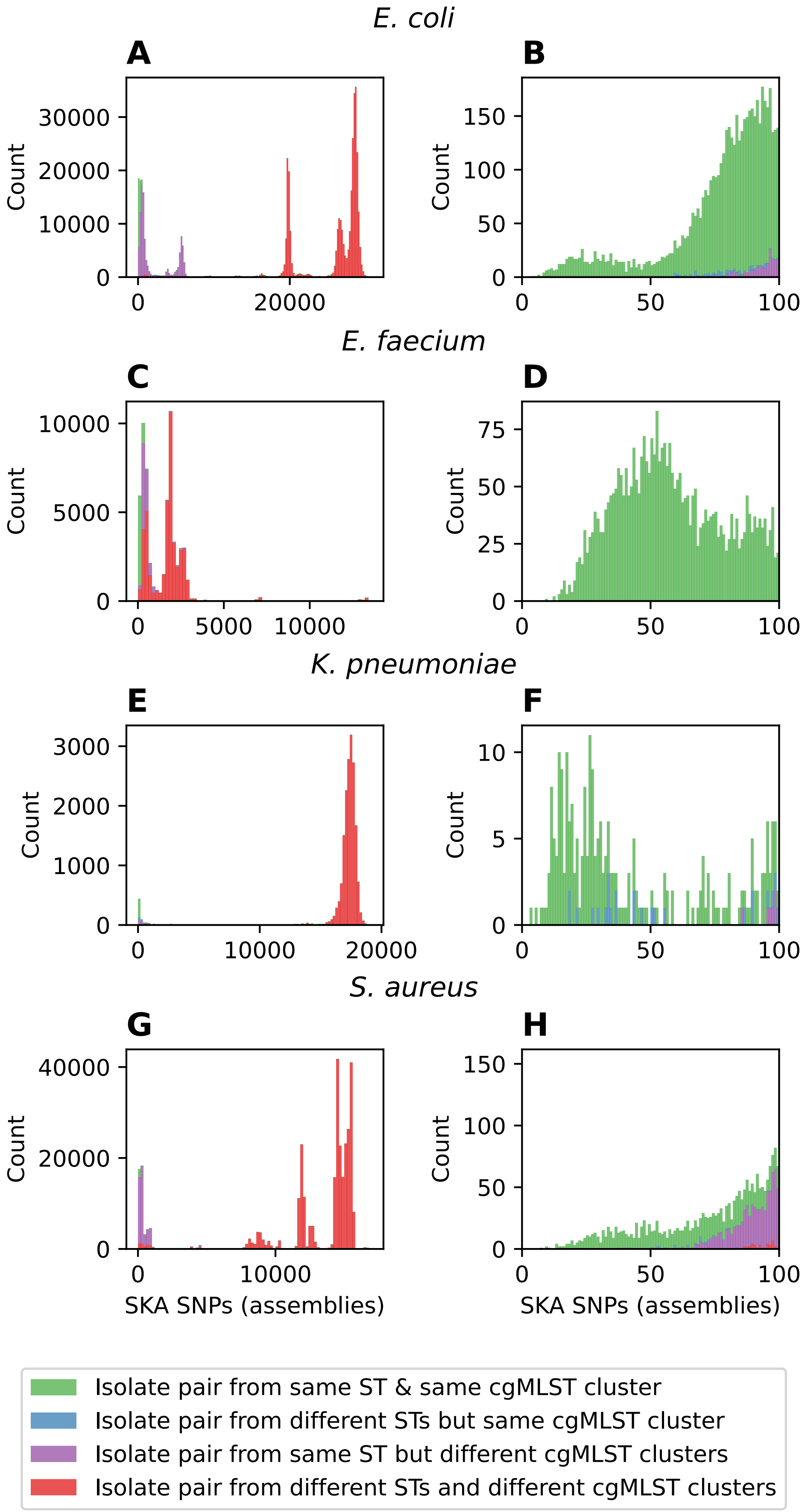

E. coli
A
B
E. faecium
C
D
K. pneumoniae
E
F
S. aureus
G
H
Count
SKA SNPs (assemblies)
Isolate pair from same ST & same cgMLST cluster
Isolate pair from different STs but same cgMLST cluster
Isolate pair from same ST but different cgMLST clusters
Isolate pair from different STs and different cgMLST clusters

Figure S17. Frequency distribution of SNP distances calculated with SKA using genome assemblies as input (fasta). The SNP distance between a pair of isolates (x axis) is plotted against the frequency with which that distance occurs (y axis). The distributions are coloured based upon whether the pair of isolates is from the same ST and cgMLST cluster (single linkage clustering, 25 allelic differences) (green), different ST and same cgMLST cluster (blue), same ST and different cgMLST cluster (purple) or different ST and different cgMLST cluster (red). Four bacterial species are shown (*E. coli* A and B, *E. faecium* C and D, *K. pneumoniae* E and F, and *S. aureus* G and H). The full distributions are shown in panels A, C, E and G. The distribution up to a SNP distance of 100 SNPs is shown in panels B, D, F and H. Small SNP distances, (indicative of closely related isolates) measured by SKA are from the same ST and cgMLST cluster, consistent with existing outbreak identification methods.

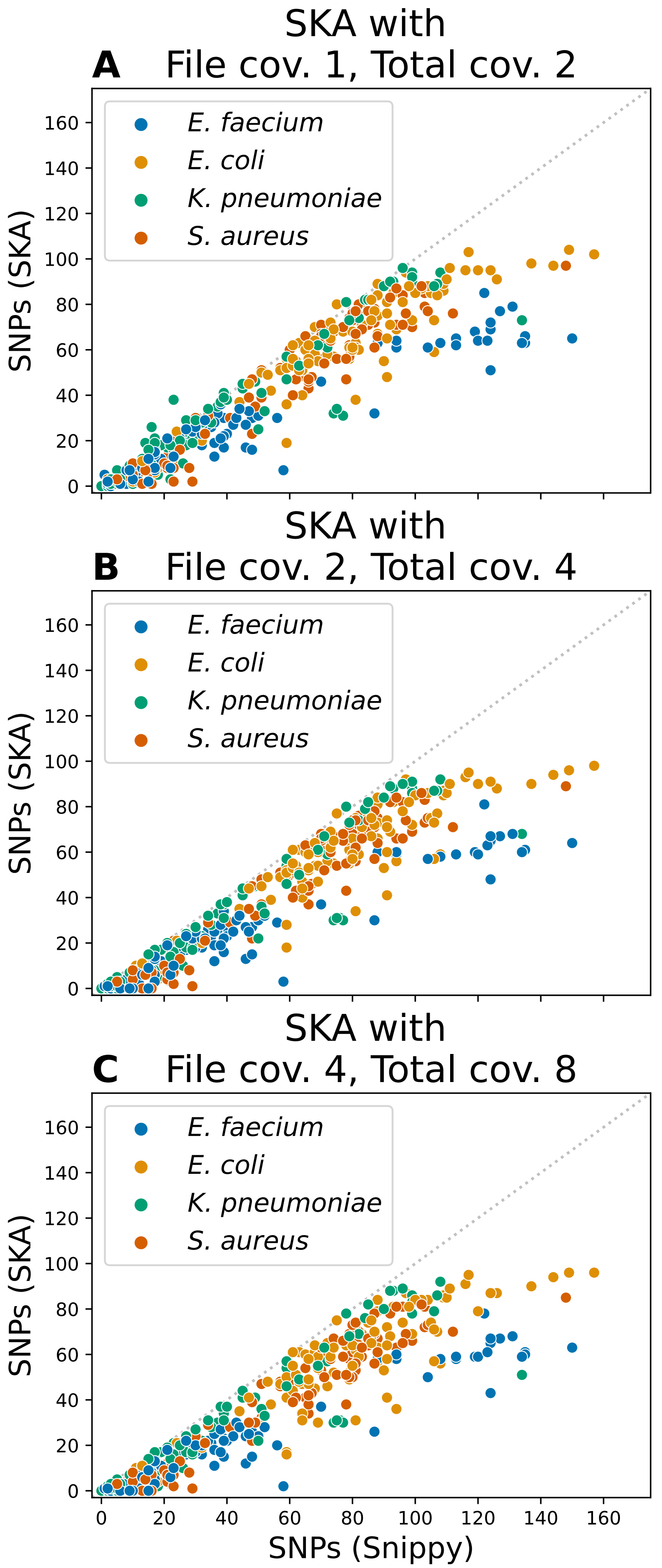

A
SKA with
File cov. 1, Total cov. 2
B
SKA with
File cov. 2, Total cov. 4
C
SKA with
File cov. 4, Total cov. 8
E. faecium
E. coli
K. pneumoniae
S. aureus
SNPs (SKA)
SNPs (Snippy)
0
20
40
60
80
100
120
140
160

Figure S18. Comparison of SKA with Snippy. One hundred real-world isolates with a SKA distance of less than 100 SNPs were chosen at random for pairwise analysis with Snippy. SNPs detected with SKA (y axis) using sequencing reads, a k-mer length of 19 and MAF filter of 0.01. K-mer coverage cutoffs of 1 file & 2 total (A), 2 file & 4 total (B) and 4 file & 8 total (C) are plotted against SNPs detected by Snippy (x axis). Points are coloured by bacterial species with *E. faecium* in blue, *E. coli* in gold, *K. pneumoniae* in green and *S. aureus* in red. Dotted grey line indicates equality between Snippy and SKA. When using file coverage cutoff of 1 and total coverage cutoff of 2 resulted in 18 out 400 instances where SKA reported more SNPs than Snippy (maximum discrepancy of 15 SNPs). Use of file coverage cutoff of 2 and total coverage cutoff of 4 resulted in only 2 out of 400 instances where SKA reported more SNPs than Snippy (maximum discrepancy of 2 SNPs), while using coverage cutoffs of 4 and 8 resulted in 0 instances where SKA reported more SNPs than Snippy.

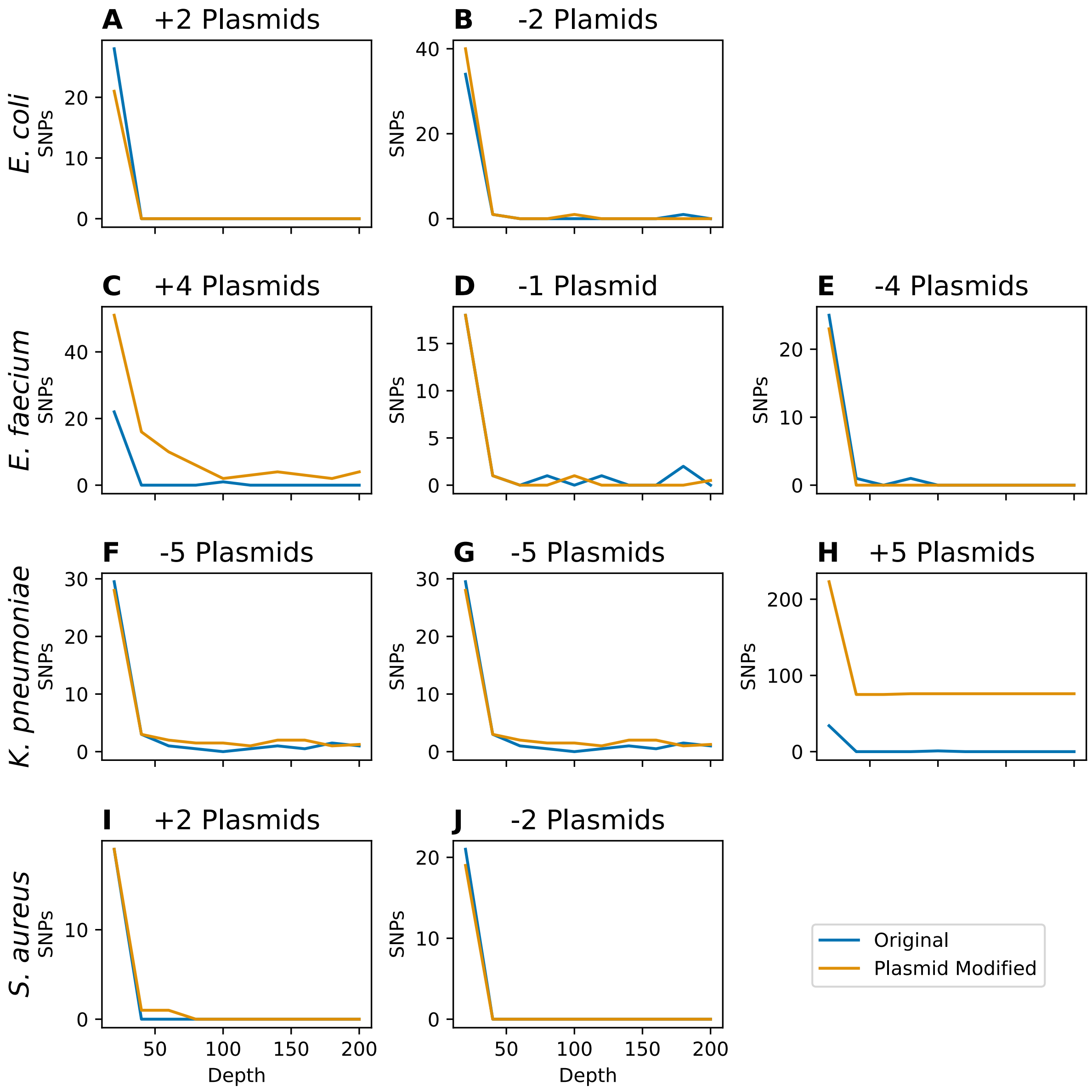

Figure S18. Impact of simulated plasmid movement on the detection of SNPs with SKA-fasta. Number of SNPs detected with SKA (y axis) against depth of sequencing (x axis). In blue, the original unmodified, re-assembled genome was compared to itself as a negative control. Detection of SNPs likely arises due to the genome assembler. In orange, the plasmid modified assembly is shown. See table 3 for details of chromosome and plasmid combinations.
